# Lineage‐specific plastid degradation in subtribe Gentianinae (Gentianaceae)

**DOI:** 10.1002/ece3.7281

**Published:** 2021-02-22

**Authors:** Peng‐Cheng Fu, Shan‐Shan Sun, Alex D. Twyford, Bei‐Bei Li, Rui‐Qi Zhou, Shi‐Long Chen, Qing‐Bo Gao, Adrien Favre

**Affiliations:** ^1^ School of Life Science Luoyang Normal University Luoyang China; ^2^ Ashworth Laboratories Institute of Evolutionary Biology The University of Edinburgh Edinburgh UK; ^3^ Royal Botanic Garden Edinburgh Edinburgh UK; ^4^ Key Laboratory of Adaptation and Evolution of Plateau Biota Northwest Institute of Plateau Biology Chinese Academy of Sciences Xining China; ^5^ Qinghai Provincial Key Laboratory of Crop Molecular Breeding Xining China; ^6^ Senckenberg Research Institute and Natural History Museum Frankfurt am Main Germany

**Keywords:** divergence dating, molecular evolution, *ndh* complex, plastome, substitution rate, subtribe Gentianinae

## Abstract

The structure and sequence of plastid genomes is highly conserved across most land plants, except for a minority of lineages that show gene loss and genome degradation. Understanding the early stages of plastome degradation may provide crucial insights into the repeatability and predictability of genomic evolutionary trends. We investigated these trends in subtribe Gentianinae of the Gentianaceae, which encompasses ca. 450 species distributed around the world, particularly in alpine and subalpine environments. We sequenced, assembled, and annotated the plastomes of 41 species, representing all six genera in subtribe Gentianinae and all main sections of the species‐rich genus *Gentiana* L. We reconstructed the phylogeny, estimated divergence times, investigated the phylogenetic distribution of putative gene losses, and related these to substitution rate shifts and species’ habitats. We obtained a strongly supported topology consistent with earlier studies, with all six genera in Gentianinae recovered as monophyletic and all main sections of *Gentiana* having full support. While closely related species have very similar plastomes in terms of size and structure, independent gene losses, particularly of the *ndh* complex, have occurred in multiple clades across the phylogeny. Gene loss was usually associated with a shift in the boundaries of the small single‐copy and inverted repeat regions. Substitution rates were variable between clades, with evidence for both elevated and decelerated rate shifts. Independent lineage‐specific loss of *ndh* genes occurred at a wide range of times, from Eocene to Pliocene. Our study illustrates that diverse degradation patterns shape the evolution of the plastid in this species‐rich plant group.

## Graphical abstract

This study traces plastome evolution of all main lineages of the species‐rich group Gentianinae, and shows lineage‐specific patterns of plastid degradation and shifts in substitution rates.

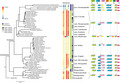

## 1 INTRODUCTION

The increasing availability of plastid genomes represents a new opportunity to explore molecular evolution in plants (Tonti‐Filippini et al., 2017; Twyford & Ness, 2017). For example, plastid phylogenomics has resolved some persistent taxonomic uncertainties in challenging plant groups (e.g., in Rosaceae; Zhang et al., 2017), and more generally led to a better understanding of major events in plant evolution (e.g., the consequences of the Jurassic gap; Li et al., 2019). Furthermore, comparing plastome structure among related clades and linking the structural changes with substitution rates can offer clues to the mechanisms driving their evolution.

In land plants, plastid genomes are usually composed of two inverted repeat (IR) regions that are separated by the large single‐copy (LSC) region and the small single‐copy (SSC) region (Jansen & Ruhlman, 2012). Comparative analysis among closely related taxa can provide insights into the microstructural evolution of plastid genomes (Mower & Vickrey, 2018), including IR expansion/reduction (Choi et al., 2019; Weng et al., 2017), sequence inversion (Mower et al., 2019), and gene loss (Graham et al., 2017; Song et al., 2017; Yao et al., 2019). Plastome microstructural change can also provide synapomorphies to support sequence‐based phylogenetic results, as seen with the loss of the *rpl2* intron in Asteropeiaceae and Physenaceae (Yao et al., 2019). Furthermore, some trends in the evolution of plastome structure are known to be associated with changes in nucleotide substitution rates (Weng et al., 2017) and selection pressures (Wicke et al., 2016). Elevated substitution rates are often associated with changes in plastome size (Schwarz et al., 2017) or in life‐history (Gaut et al., 2011). Some remarkable modifications of plastid genomes have been observed in nonphotosynthetic parasites (e.g., holoparasitic members of the Orobanchaceae). In these species, the functional loss of photosynthetic genes correlates with microstructural changes and accelerated substitution rates due to relaxed selection, resulting in miniaturized plastid genomes with a greatly reduced gene content (Wicke et al., 2016). This plastid genome degradation starts with small scale losses of nonessential genes and the accumulation of microstructural changes, followed by further phases of elevated evolution and gene losses on a trajectory of reductive plastome evolution.

The study of *ndh* (NADH dehydrogenase‐like) genes has provided many useful insights into gene loss, gene degradation, and gene retention in plants. The *ndh* genes produce the NADH complex, which is essential for electron cycling in photosystem I under heat‐stressed conditions (Wang et al., 2006). Since *ndh* is often the first gene family to be lost in the process of plastid degradation (Mohanta et al., 2020), studying it contributes to our understanding of the early stages of degradation that are likely to occur in many plant lineages. Eleven *ndh* genes are present in nearly all flowering plant species studied to date, as compared to 150–200 in the cyanobacterial plastid ancestors. *ndh* genes have been lost in nonphotosynthetic parasites due to a relaxation of selective constraints (Barrett et al., 2014; Wicke et al., 2016), but independent losses have also occurred in a minority of photosynthetic plant lineages (Mohanta et al., 2020; Ruhlman et al., 2015), such as in Gnetales and other conifers (Braukmann et al., 2009), Alismatales (Ross et al., 2016), orchids (Kim et al., 2019; Lin et al., 2017), and Geraniaceae (Ruhlman & Jansen, 2018). *Ndh* may be uniformly lost in a lineage or show a more dynamic fate with presence/absence (or pseudogenization) among populations or closely related species (Barrett et al., 2018; Kim et al. 2019). Overall, the fate of *ndh* genes appears to be complex, as their conservation across most flowering plants suggests a strong selective advantage for their retention, yet their repeated loss and dispensability under benign nonstressful conditions suggests otherwise (Martín & Sabater, 2010; Ruhlman et al., 2015; Wang et al., 2006). Many cases of plastid gene loss can be explained by transfer of functional copies to the nuclear genome (Kleine et al., 2009; Liu et al., 2020; Martin et al., 1998), and this may also be expected for *ndh* genes. Loss of *ndh* genes has usually been observed from sparse taxon sampling, as is the case in the genus *Gentiana* L. (Sun et al., 2018). As such, we have a limited understanding of the phylogenetic distribution of gene losses and the selection pressures involved in this loss of *ndh* genes in this genus and many others.

The family Gentianaceae, and in particularly *Gentiana*, have long attracted the attention of scientists because of their medical, chemical, and horticultural value (Ho & Liu, 2001; Rybczyński et al., 2015). *Gentiana* species are predominantly alpine and occur in numerous mountain systems around the world (Ho & Liu, 2001). Biogeographic studies have shown that the Qinghai–Tibet Plateau (QTP) acted as the primary source area for *Gentiana* to disperse to many other distant mountainous areas, and is the center of biodiversity for these species (Favre et al., 2016). Although our understanding of the taxonomy and phylogenetic relationships within *Gentiana* and subtribe Gentianinae has greatly improved in the past two decades, little is known about patterns, trends, and modes of molecular evolution among Gentianinae genera and sections within *Gentiana*. For example, plastome sequences of species from section *Kudoa* (Masamune) Satake & Toyokuni ex Toyokuni have revealed contrasting patterns of plastome sequence evolution, with some but not all species showing notable plastome size decreases and *ndh* gene losses (Fu et al., 2016; Sun et al., 2018). In contrast, only subtle sequence divergence and microstructural change are present among species in three other sections (Ni et al., 2017; Sun Wang & Fu, 2019; Sun, Zhou, et al., 2019; Zhou et al., 2018). Based on these results, *Gentiana* and its closely related genera appear to be a promising system for investigating plastome evolution and its link to evolutionary transitions, such as in life‐history. Indeed, most species of subtribe Gentianinae are perennials though there are a few clades of annuals, that are characterised by long branches in phylogenetic analysis (Favre et al., 2016) and likely subject to rapid evolution (Yuan & Küpfer, 1997).

In this study, we aim to investigate plastome evolution over ca. 40 million years (Gentianinae stem age; Favre et al., 2016) using all main extant lineages of Gentianinae, including all genera and almost all currently accepted sections of *Gentiana*. We aim to relate diversity in plastome structure to phylogeny and species’ attributes such as life‐history and habitat. We sequence, assemble, and annotate the complete plastid genomes of 41 species, and integrate these with existing plastome data. We use these data to assess whether: (a) there is repeated independent losses of *ndh* and other genes across the phylogeny of subtribe Gentianinae, and (b) plastome gene loss is associated with biological traits (such as life‐history) or other factors such as shifts in evolutionary rates. These results from plastomes will provide crucial insights into the predictability of gene losses and the lability in plastid genome structure. Moreover, by sampling across the diversity of subtribe Gentianinae, we can identify the generalities and the idiosyncratic changes in the early stages of plastome restructuring.

## 2 MATERIALS AND METHODS

### 2.1 Taxon sampling

A total of 41 species were sampled representing all six genera in subtribe Gentianinae and all currently accepted sections of *Gentiana* (Favre et al., 2014, 2020; Appendix A1) except sect. *Tetramerae*, which is species‐poor and was newly established (Favre et al., 2020). Samples of leaves (for large perennials or annuals) or whole plant (minute annuals) were collected in the wild from a single plant for each species and dried in silica gel. Species were identified by Dr. Peng‐Cheng Fu and Dr. Adrien Favre, and their voucher specimens were deposited either in the herbarium of Luoyang Normal University (no acronym at present), Frankfurt am Main (Herbarium Senckenbergianum, FR), Leipzig (LZ), or in Kunming (KUN).

### 2.2 Plastid genome sequencing, assembly, and annotation

Total genomic DNA isolation, DNA fragmentation, and sequencing library construction followed the methodology described in Fu et al. (2016). The genomic DNA library of each species was sequenced using the Illumina HiSeq 2500 platform (Novogene), with 150‐bp paired‐end reads. Raw reads were filtered and trimmed with Trimmomatic v0.32 (Bolger et al., 2014) with default parameters to remove adaptor sequences and low‐quality reads and sites, and then checked for quality with FastQC v0.11.2 (https://www.bioinformatics.babraham.ac.uk/projects/fastqc/). Plastomes were assembled de novo using NOVOPlasty 2.6.1 (Dierckxsens et al., 2016) with a k‐mer size of 39 bp. Each plastid genome was annotated with GeSeq (Tillich et al., 2017) and PGA (Qu et al., 2019) using the default parameters. Geneious Basic 5.6.4 (Kearse et al., 2012) was used to manually check and modify annotations. All plastome sequences were saved as GB2sequin files (Lehwark & Greiner, 2018) and deposited in GenBank (Table 1). We verified one large insertion of 5 kB found in *G. cuneibarba* (see Section 3) using custom primers (Supplementary A1). Three PCRs were performed to verify the boundaries, as well as the middle insert sequence. PCR products were then sent for Sanger sequencing. The insertion sequence was annotated using BlastN with default settings.

**TABLE 1:** Plastome structure and sequence information for Gentianaceae species included in this study. Columns LSC, IR and SSC report the length of the large single‐copy, inverted repeat and small single‐copy regions, respectively, in base pairs

Species	Taxonomic treatment	NCBI no.	LSC	IR	SSC	Total
*Gentiana altigena*	sect. *Kudoa* ser. *Ornatae*	MN234140*	77,727	23,596	12,336	137,255
*Gentiana caelestis*	sect. *Kudoa* ser. *Ornatae*	MG192304	77,870	24,113	11,548	137,644
*Gentiana dolichocalyx*	sect. *Kudoa* ser. *Ornatae*	MN199161*	77,918	24,560	10,491	137,529
*Gentiana futtereri*	sect. *Kudoa* ser. *Ornatae*	MN199155*	77,939	23,864	11,823	137,490
*Gentiana lawrencei*	sect. *Kudoa* ser. *Ornatae*	KX096882	78,082	24,635	11,365	138,750
*Gentiana obconica*	sect. *Kudoa* ser. *Ornatae*	MG192306	77,754	23,865	11,794	137,278
*Gentiana oreodoxa*	sect. *Kudoa* ser. *Ornatae*	MG192307	77,908	23,865	11,765	137,403
*Gentiana ornata*	sect. *Kudoa* ser. *Ornatae*	MG192308	77,816	24,108	11,353	137,385
*Gentiana veitchiorum*	sect. *Kudoa* ser. *Ornatae*	MG192310	77,932	23,864	11,807	137,467
*Gentiana hexaphylla*	sect. *Kudoa* ser. *Verticillatae*	MG192305	77,922	23,865	11,771	137,423
*Gentiana ternifolia*	sect. *Kudoa* ser. *Verticillatae*	MN199147*	77,762	24,090	11,574	137,516
*Gentiana tetraphylla*	sect. *Kudoa* ser. *Verticillatae*	MN199152*	77,926	23,831	11,822	137,410
*Gentiana viatrix*	sect. *Kudoa* ser. *Verticillatae*	MN199159*	77,925	23,831	11,822	137,409
*Gentiana georgei*	sect. *Isomeria* ser. *Monanthae*	MN234135*	81,586	25,421	16,926	149,354
*Gentiana stipitata*	sect. *Isomeria* ser. *Monanthae*	MG192309	79,712	25,229	16,986	147,156
*Gentiana szechenyii*	sect. *Isomeria* ser. *Monanthae*	MN199158*	81,581	25,387	16,979	149,334
*Gentiana cephalantha*	sect. *Monopodiae* ser. *Apteroidea*	MN199135*	79,373	25,237	17,026	146,873
*Gentiana davidii*	sect. *Monopodiae* ser. *Apteroidea*	MN199156*	79,945	25,277	17,066	147,565
*Gentiana sikkimensis*	sect. *Monopodiae* ser. *Sikkimenses*	MN199154*	79,370	24,850	17,033	146,103
*Gentiana wardii*	sect. *Monopodiae* ser. *Sikkimenses*	MN234136*	79,357	25,191	15,604	145,343
*Gentiana crassicaulis*	sect. *Cruciata*	KJ676538	81,164	25,271	17,070	148,776
*Gentiana cruciata*	sect. *Cruciata*	MN199136*	81,221	25,310	17,092	148,933
*Gentiana dahurica*	sect. *Cruciata*	MH261259	81,154	25,278	17,093	148,803
*Gentiana hoae*	sect. *Cruciata*	MN199141*	81,266	25,321	17,084	148,992
*Gentiana macrophylla*	sect. *Cruciata*	KY856959	82,911	24,955	17,095	149,916
*Gentiana officinalis*	sect. *Cruciata*	MH261261	81,119	25,336	17,088	148,879
*Gentiana robusta*	sect. *Cruciata*	KT159969	81,164	25,333	17,081	148,991
*Gentiana siphonantha*	sect. *Cruciata*	MH261260	81,121	25,337	17,113	148,908
*Gentiana straminea*	sect. *Cruciata*	KJ657732	81,240	25,333	17,085	148,991
*Gentiana tibetica*	sect. *Cruciata*	KU975374	81,163	25,266	17,070	148,765
*Gentiana atuntsiensis*	sect. *Frigida*	MN199151*	77,276	24,980	17,001	144,237
*Gentiana handeliana*	sect. *Frigida*	MN199143*	77,014	24,917	16,965	143,813
*Gentiana nubigena*	sect. *Frigida*	MN199157*	77,439	24,700	16,539	143,378
*Gentiana trichotoma*	sect. *Frigida*	MN089577	77,430	25,162	17,005	144,759
*Gentiana phyllocalyx*	sect. *Phyllocalyx*	MN199146*	73,079	30,066	2,352	135,563
*Gentiana yunnanensis*	sect. *Microsperma* ser. *Suborbisepalae*	MN199140*	79,734	25,444	16,839	147,461
*Gentiana tongolensis*	sect. *Microsperma* ser. *Suborbisepalae*	MK251985	78,289	25,359	16,750	145,757
*Gentiana lutea*	sect. *Gentiana*	MN199129*	81,815	25,700	17,251	150,466
*Gentiana purpurea*	sect. *Gentiana*	MN199160*	81,791	25,758	17,251	150,558
*Gentiana bavarica*	sect. *Calathianae*	MN199162*	80,232	25,468	16,726	147,894
*Gentiana terglouensis*	sect. *Calathianae*	MN199132*	80,184	25,468	16,720	147,840
*Gentiana acaulis*	sect. *Ciminalis*	MN199148*	81,870	25,675	17,231	150,451
*Gentiana clusii*	sect. *Ciminalis*	MN199142*	80,734	25,566	17,301	149,167
*Gentiana scabra*	sect. *Pneumonanthe*	MN199131*	81,350	25,285	17,269	149,189
*Gentiana haynaldii*	sect. *Chondrophylla* ser. *Dolichocarpa*	MN234137*	73,530	22,121	10,117	127,889
*Gentiana producta*	sect. *Chondrophylla* ser. *Dolichocarpa*	MN199163*	70,075	19,878	7,949	117,780
*Gentiana aristata*	sect. *Chondrophyllae* ser. *Humiles*	MN234139*	73,698	22,355	9,367	127,775
*Gentiana crassuloides*	sect. *Chondrophyllae* ser. *Orbiculatae*	MN199150*	73,203	22,370	10,449	128,392
*Gentiana cuneibarba*	sect. *Chondrophylla* ser. *Fimbricorona*	MN199137*	73,493	22,460	15,164	133,577
*Kuepferia damyonensis*	*Kuepferia*	MN199133*	78,521	23,789	16,795	142,894
*Kuepferia decorata*	*Kuepferia*	MN199130*	77,771	22,004	15,022	136,801
*Metagentiana gentilis*	*Metagentiana*	MN199138*	79,277	25,720	17,614	148,331
*Metagentiana rhodantha*	*Metagentiana*	MN199153*	79,926	25,762	17,637	149,087
*Sinogentiana souliei*	*Sinogentiana*	MN234138*	74,329	24,177	11,653	134,336
*Sinogentiana striata*	*Sinogentiana*	MN199149*	78,009	24,669	16,935	144,282
*Crawfurdia campanulacea*	*Crawfurdia*	MN199164*	81,123	25,685	17,595	150,088
*Crawfurdia poilanei*	*Crawfurdia*	MN199145*	81,854	25,677	17,386	150,594
*Tripterospermum championii*	*Tripterospermum*	MN199139*	82,506	25,602	17,640	151,350
*Tripterospermum luteoviride*	*Tripterospermum*	MN199144*	82,177	25,584	17,584	150,929
*Swertia bimaculata*	*Swertia*	MH394372	84,588	25,436	18,342	153,802
*Swertia mussotii*	*Swertia*	KU641021	83,567	25,761	18,342	153,431
*Swertia verticillifolia*	*Swertia*	MF795137	82,623	25,362	18,335	151,682

Newly sequenced plastomes are marked with asterisks next to the GenBank accession numbers.

### 2.3 Phylogenetic analysis

To establish phylogenetic relationships among lineages, we used our 41 newly sequenced plastomes, in addition to 18 previously published plastomes in subtribe Gentianinae available from GenBank (Table 1). Four species with available plastomes in subtribe Swertiinae were used as outgroups. Sequences of all protein‐coding genes were extracted in PhyloSuite (Zhang et al., 2020) and aligned using MAFFT (Katoh et al., 2002). A protein‐coding matrix was constructed where we excluded genes that were absent in some species or that showed variability that made alignment difficult. We examined the matrix and removed the most rapidly evolving sites using Gblocks (Talavera & Castresana, 2007) using default setting. Phylogenetic analyses were performed with IQ‐TREE (Nguyen et al., 2014) implemented in PhyloSuite (Zhang et al., 2020) using maximum likelihood (ML) and with 1,000 rapid bootstrap replicates. The substitution model was chosen using ModelFinder (Kalyaanamoorthy et al., 2017). The trees were visualized and edited using FigTree 1.4.0 (http://tree.bio.ed.ac.uk/software/Figtee/).

### 2.4 Divergence dating

We estimated the divergence times of main lineages using the Bayesian method implemented in BEAST 2.4 (Bouckaert et al., 2014; Drummond et al., 2012). We ran the analyses using the HKY substitution model, the Yule model, and the strict clock model. The stem node of *G*. sect. *Cruciata* was constrained with a taxonomically unambiguous fossil (Mai, 2000; Mai & Walther, 1988), which was originally considered to be from the Pliocene (Mai & Walther, 1988), but is now believed to be from the early Miocene (Mai, 2000). We used lognormal priors with an offset at 16.0 Ma, a mean of 1, and a standard deviation of 1.0. To improve the accuracy of the molecular dating given the very limited fossil evidence available for gentians, we also constrained the crown age of *Gentiana* based on the date from a broad‐scale molecular phylogenetic analysis of the Angiosperms. We used uniform priors (Schenk & Axel, 2016) with a lower age of 21.25 Ma and an upper age of 38.21 Ma to encompass the entire 95% highest posterior density (HPD) dates from Janssens et al. (2020). We also performed an analysis of priors only (without sequence data) to check whether there was prior interaction (Warnock et al., 2015). We ran three independent MCMC chains with 10 million generations, sampling every 1,000th generation and discarding the initial 10% as burn‐in. Convergence was confirmed in TRACER 1.5 (http://tree.bio.ed.ac.uk/software/tracer/) and judged as suitable by ESS values (>200). Trees were summarized using TreeAnnotator 1.7.5 (Drummond et al., 2012).

### 2.5 Plastome microstructural changes

Genome comparisons were conducted to identify structural differences among the 59 taxa included in this study, using mVISTA (Frazer et al., 2004) and Geneious Basic 5.6.4 (Kearse et al., 2012). We characterized structural changes for each species as the number of discrete events (where an event is a protein‐coding gene loss, intron loss, or pseudogenization). Genes on the boundaries of the junction sites were visualized in IRscope (Amiryousefi et al., 2018). We analyzed rearrangement histories by using the progressive Mauve algorithm in Mauve v2.3.1 (Darling et al., 2010) using the plastid genome sequence with only one IR copy. To visualize gene losses across the phylogeny, we labeled branches where all taxa in a given clade had lost a particular gene. We tested whether boundary shifts (LSC‐IR and SSC‐IR) and plastome size changes have phylogenetic signal using Pagel's lambda (Pagel, 1997, 1999) in the R package *MOTMOT* (Puttick et al., 2020).

### 2.6 Nucleotide substitution rate

Nonsynonymous (dN) and synonymous (dS) nucleotide substitution rates were analyzed with HyPhy 2.2 (Pond et al., 2005). Given the strong similarities among species within sections of *Gentiana* or within other genera (see Results), a maximum of five species per clade were chosen for these analyses (Appendix A2). Codon‐wise alignments of each gene were performed in Geneious Basic 5.6.4 (Kearse et al., 2012). *Rps16* was excluded due to the presence of reading frame shifts caused by pseudogenization across the phylogeny (see Results). Tests of relative dN and dS for all plastid protein genes were carried out by using the MG94 × REV model with a corrected 3 × 4 codon frequency estimator, using a guide tree generated in this study (see above). The Pearson correlation coefficient (*r*) between average substitution rates and total plastome sizes, and their significances were calculated.

## 3 RESULTS

### 3.1 General plastome characteristics

**FIGURE 1:**
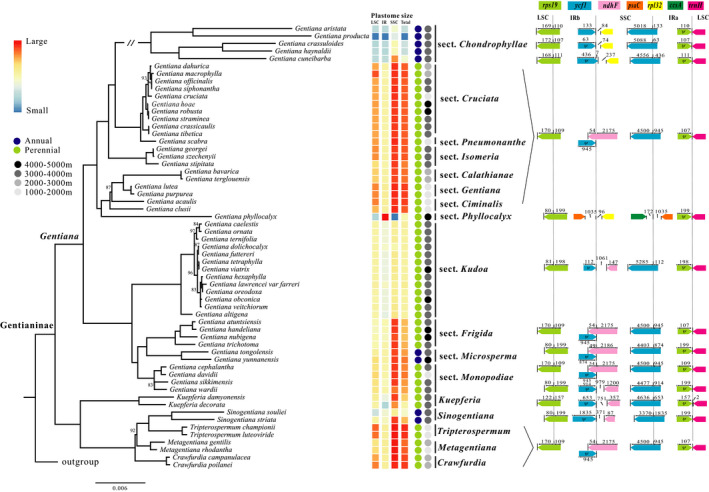
Phylogenetic topology and plastid boundary changes in subtribe Gentianinae. The topology is derived from an analysis of plastid protein‐coding genes. All nodes have 100% bootstrap support in maximum likelihood analyses, except those indicated. Heatmaps illustrate changes in plastid size (LSC, IR, SSC, and total) with smaller in blue and larger in red. Detailed boundary structure of each newly sequenced plastome are presented in Appendix B2

The complete plastomes of 41 species of subtribe Gentianinae were successfully sequenced and assembled. We detected substantial length variation among plastomes, with total plastome size varying from 117,780 to 151,350 bp, and with size differences in the LSC (70,075–82,911 bp), IR (19,878–30,066 bp), and SSC (2,352–17,640 bp) (Table 1). Generally, species from the same section or genus had very similar plastome sizes (Figure 1). Section *Chondrophyllae* *sensu* *lato* had smaller plastomes (in terms of the LSC, IR, SSC, and total size) than other groups. Significant modifications were found in sect. *Phyllocalyx*, which had an extremely short SSC region (2,352 bp) and a long IR region (30,066 bp). Other clades, such as sect. *Kudoa*, sect. *Monopodiae* (*G. wardii*), and *Kuepferia* (*K. decorata*), also had smaller plastomes than their closely related taxa, mostly because of variation in SSC length (Table 1).

### 3.2 Phylogenetic relationship and divergence times

After filtering, the phylogenetic data matrix included 64 protein‐coding genes shared among all samples. Phylogenetic analyses based on this matrix resulted in a strongly supported topology of subtribe Gentianinae (Figure 1). Most nodes, except a few within sections, were fully supported (bootstrap support value, BS = 100) (Figure 1). The topology is identical to Favre et al. (2014, 2016) at the generic level and Favre et al. (2020) at the sectional level. We found that subtribe Gentianinae was divided into two clades. The first included five strongly supported genera (BS = 100). The second corresponded to *Gentiana*, which was further divided into two main subclades. All sampled sections in *Gentiana* were monophyletic with full support (BS = 100) (Figure 1).

**FIGURE 2:**
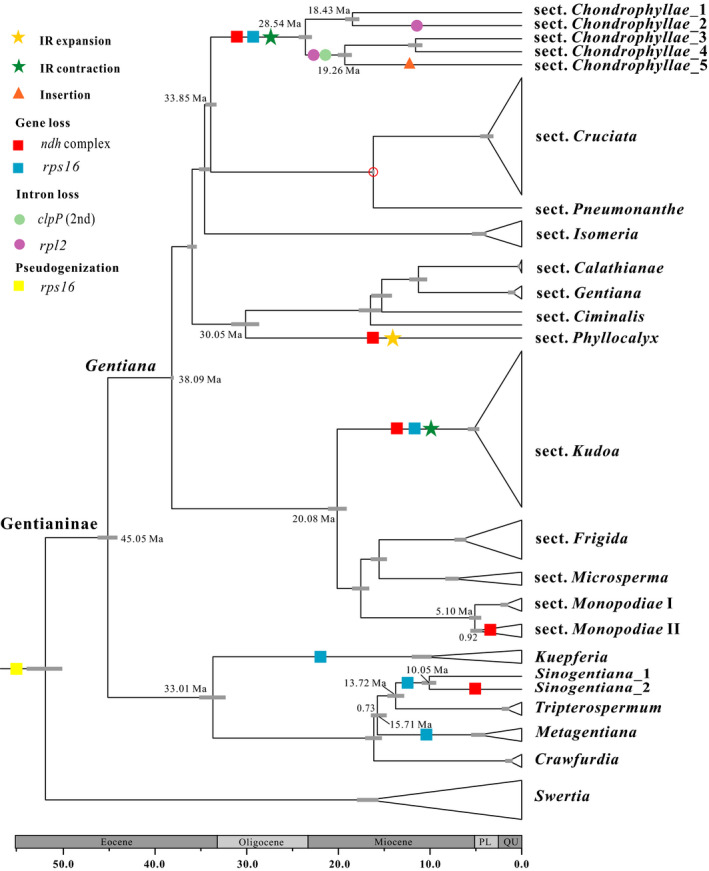
Divergence dating and plastid structural changes (loss of protein‐coding genes, loss of introns, sequence insertions, IR expansion and contraction) in subtribe Gentianinae. The gray bars show the 95% highest posterior density on the age estimates. The red circle shows the node constrained with a fossil calibration. Ma, million years ago; PL, Pliocene; QU, quaternary

Divergence time analysis without sequence data showed that the effective calibration priors in the fossil and secondary nodes were 21.96 Ma (95% HPD: 16.50–29.07 Ma) and 30.50 Ma (95% HPD: 22.91–38.20 Ma), respectively, demonstrating that the specified calibration priors are faithfully implemented in the joint estimation of divergence times. Our subsequent divergence time analyses showed that the crown age in Gentianinae and *Gentiana* was 45.05 Ma (95% HPD: 44.01–46.16 Ma) and 38.09 Ma (95% HPD: 37.88–38.21 Ma), respectively (Figure 2). We recovered age estimates for a range of key lineages, including those with microstructural changes (discussed below), with *Chondrophyllae s. l*., *Phyllocalyx*, *Kudoa*, and *Monopodiae* II diverging from their sister clades 33.85 Ma (95% HPD: 33.22–34.45 Ma), 30.05 Ma (95% HPD: 28.572–31.62 Ma), 20.08 Ma (95% HPD: 19.05–21.10 Ma), and 5.10 Ma (95% HPD: 4.40–5.72 Ma), respectively. Divergence within *Sinogentiana* occurred 10.05 Ma (95% HPD: 9.30–10.88 Ma).

### 3.3 Plastome microstructural changes

We found that closely related species (within a section or a genus) had very similar plastome structure. One gene complex (*ndh*) and two introns (*clpP* 2nd intron, *rpl2* intron) have been fully or partly lost in several clades (Figure 2, Supplementary B1). For instance, the two introns were lost only in some species of sect. *Chondrophyllae s. l*. The *ndh* complex of 11 plastid genes has been fully or partly lost along with the flanking regions (Supplementary B1) in five different sections or genera. *Rps16* was pseudogenized across the phylogeny and has been completely lost in some lineages (Figure 2). In *rps16*, parts (or whole) of exon 1, exon 2, or the intron were lost in most species, and the gene was completely lost in sect. *Chondrophyllae s. l*., sect. *Kudoa*, *Kuepferia*, *Sinogentiana*, and *Metagentiana*. In addition, one insertion of about 5 kB (Supplementary A1), located between *cssA* and *ndhD*, was detected in *G. cuneibarba* (sect. *Chondrophyllae s*. *l*.). The top 100 sequence matches for this insertion based on BLAST searches are presented in Dryad (https://doi.org/10.5061/dryad.h70rxwdgw). No annotated gene was present in the insertion, though one fragment had sequence similarity to plant mitochondrial genomes (e.g., *Sesuvium portulacastrum*, MN683736; bit scores and *e*‐value are 784 and 0, respectively), hinting to an origin via intergenomic transfer as in several reported cases (Burke et al., 2018; Ma et al., 2015; Raman et al., 2019). In summary, most plastome changes occurred in five phylogenetically distinct sections or genera (*Sinogentiana*, *G*. sects. *Phyllocalyx*, *Kudoa*, *Monopodiae*, and *Chondrophyllae s*. *l*.).

In subtribe Gentianinae, an expansion of the IR (represented by a yellow star in Figure 2) was observed in sect. *Phyllocalyx*. This expansion was due to the duplication of seven plastid genes (*rps15*, *ndhH*, *ndhI*, *ndhG*, *ndhE*, *psaC*, and *ndhD*; Appendix B1), yielding a 4.5 kB increase in IR length compared with its most closely related taxa (e.g., sect. *Ciminalis*). The boundaries between the IR and the SSC/LSC were conserved within sections or genera, except in sect. *Chondrophyllae s. l*. and sect. *Monopodiae* (Figure 1, Appendix B2).

**FIGURE 3:**
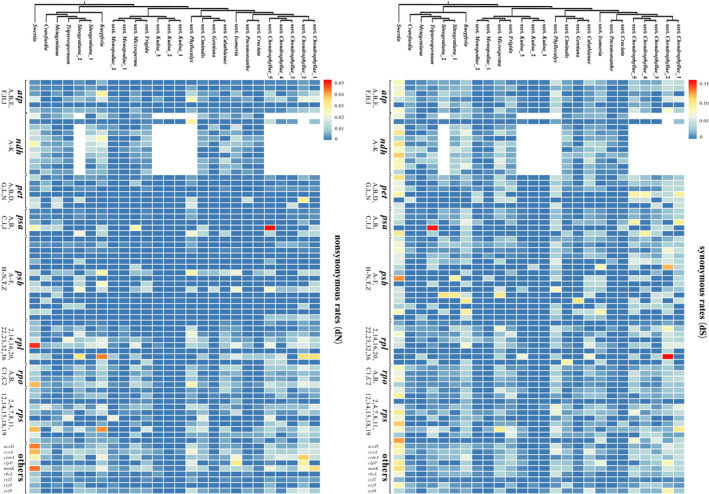
Rate variation in subtribe Gentianinae. Heatmaps illustrate differences in dN and dS for each plastid protein gene. Low rates are shown in blue, and high rates in red. The phylogenetic topology is from the maximum likelihood analysis of protein coding genes presented in Figure 1

Various junction site patterns were detected in the plastomes across subtribe Gentianinae. For example, we found one pseudogene (*ψycf1*) of variable size resulting from the incomplete duplication of the functional copy of *ycf1*. This pseudogene was common throughout subtribe Gentianinae except in sect. *Phyllocalyx*, where *ycf1* was completely duplicated (Appendix B1), and in some species of sect. *Kudoa* (*G. altigena* and *G. lawrencei*) where *ψycf1* was absent (Appendix B2). The exact location of SSC–IRb boundary varied depending on the taxon (Figure 1, Appendix B2). For example, it was in the intergenic spacer region (IGS) between *ψycf1* and *rpl32* in sect. *Chondrophyllae s. l*., whereas it was in the IGS between *psaC* and *rpl32* in sect. *Phyllocalyx*. The SSC–IRa boundary was located within *ycf1* across all species except in sections *Phyllocalyx* and *Kudoa* (*G. altigena*), where it occurred in the IGS. Finally, we found that the LSC–IR boundary was stable, but the relative position varied throughout the subtribe. In summary, species in subtribe Gentianinae had similar junction patterns, with the exception of six sections or genera (Figure 3; Appendix B2). Tests in *MOTMOT* showed that the maximum‐likelihood estimate of Pagel's lambda was equal to 1 for LSC‐IR and SSC‐IR boundary shifts and plastome size, indicating high phylogenetic signal.

### 3.4 Changes in evolutionary rate

We assessed the dN and dS values of 74 protein‐coding genes in 25 species selected to represent all sections of *Gentiana* and its allies. For most genes, both the relative dN and dS values were low, and we did not find gene classes or single genes with high relative dN and dS values in the entire subtribe (Figure 3; Supplementary A2 and A3). However, when compared among closely related clades, we found that multiple genes evolved at an elevated substitution rate in sects. *Chondrophyllae s. l*. and *Phyllocalyx*, as well as in *Kuepferia*, with higher dN and dS values in the majority of their plastid genes (incl. *rpl*, *rpo,* and *rps*). In contrast, sect. *Kudoa* showed hardly any rate shifts. Average substitution rates were significantly negatively correlated with plastome size (dN, *r* = −0.284, *p* < 0.01; dS, *r* = −0.232, *p* < 0.05).

## 4 DISCUSSION

### 4.1 Phylogeny of subtribe Gentianinae

Our study using complete plastome data generated a strongly supported topology that represents all main lineages in Gentianinae. Five closely related genera in a lineage sister to *Gentiana* were fully supported, consistent with Chen et al. (2020) and earlier studies (e.g., Favre et al., 2014). The topology within *Gentiana* in our study is very similar to those of previous studies using DNA barcoding (Favre et al., 2016) and plastid SNPs (Chen et al., 2020), and identical to a recent study that demonstrated that plastome phylogenies were identical to those using hundreds of single‐copy nuclear genes at the sectional level (Favre et al., 2020). We recovered all main lineages recognized in Favre et al. (2020) as monophyletic and further confirmed the monophyly of sections in *Gentiana*. Although comparisons with transcriptome data show some topological incongruences (Chen et al., 2020), the results from plastid data provide firm support for the relationships among major groups and acts as a useful framework phylogeny for the Gentianinae.

### 4.2 Widespread and lineage‐specific plastome gene losses in subtribe Gentianinae

The loss of plastid genes is not restricted to holoparasitic plants, but is also common in a wide range of photosynthetic vascular plants (Kim et al., 2019; Lehtonen & Cárdenas, 2019; Wicke et al., 2016; Yao et al., 2019). Our study has used dense sampling of the main lineages from subtribe Gentianinae to identify the dynamic patterns of gene losses that may occur at the early stages of plastome degradation.

Previous work on four sections of *Gentiana* (Sun et al., 2018; Sun, Zhou, et al., 2019; Zhou et al., 2018) indicated that the loss of plastid genes was seemingly specific to sect. *Kudoa*, now referred to as the monophyletic clades formed by ser. *Stragulatae*, ser. *Ornatae*, and ser. *Verticillatae* (Favre et al., 2020). However, using broader sampling covering all main lineages in this group, we find most gene losses occur repeatedly and independently at various phylogenetic depths. Assuming that the presence of these genes is the ancestral state in subtribe Gentianinae, major gene losses have happened at least four times independently across the phylogeny. For example, the *ndh* complex is absent from several clades of both annuals (e.g., sect. *Chondrophyllae s. l*.) and perennials (e.g., sect. *Kudoa*). However, the loss of some other genes appears to be heterogeneous in other sections (e.g., intron loss in sect. *Chondrophyllae s. l*.) or genera (e.g., *Sinogentiana*). For example, the 2nd intron of *clpP* has been lost in some species in *Chondrophyllae s. l*., as has previously been reported in Caryophyllales (Yao et al., 2019) and parasitic plants (Graham et al., 2017). This suggests that the evolutionary history of the annual groups (here, *Sinogentiana*, sect. *Chondrophyllae s. l*.*,* and sect. *Microsperma*) is more complex than expected, with gene losses not directly associated with life‐history strategy. Interestingly, the most frequent gene losses were found in sect. *Chondrophyllae s. l*., and phylogenetic studies based on plastid loci have revealed that branch lengths are particularly long in this clade (Favre et al., 2016). This group has higher average substitution rates (dN, 0.004; dS, 0.024) than its sister group (dN, 0.001; dS, 0.004). In fact, this group of annuals contains nearly half of all species in *Gentiana* (168 out of 350; Ho & Liu, 2001), and thus is likely to have undergone rapid diversification (Yuan & Küpfer, 1997). Nevertheless, diversification rates for *G*. sect. *Chondrophyllae s. l*. were not particularly high in a previous study by Favre et al. (2016). However, this study used BAMM (Rabosky et al., 2014) to uncover diversification rates, and this approach has recently been criticized (Moore et al., 2016). Thus, we argue that gene losses may be associated with rapid evolution in this group. In addition, our divergence dating gave a crown age for sect. *Chondrophyllae s. l*. of 28 million year ago, indicating gene losses in this section are not likely to be recent events.

Expansion or contraction of the IR region is commonly observed in angiosperms and tends to be the result of multiple genes moving either in or out of the IR (Zhu et al., 2016). In subtribe Gentianinae, the loss of genes and the subsequent size variation of plastomes seems to be associated with modifications of the structure in and around the boundaries of different regions. This was particularly the case at the IRb‐SSC boundary, since all the clades for which gene loss was detected (namely sects. *Kudoa*, *Phyllocalyx*, and *Chondrophyllae s. l*.) had variation there. This variation led to concomitant IR and SSC contractions, except for an IR expansion with an SSC contraction in sect. *Phyllocalyx*. This confirms the importance of genes located at boundaries for the structural stability of plastomes (Ruhlman & Jansen, 2018). We also detected species‐specific shifts in boundaries, for example, the IRb‐SSC boundary in *Gentiana* sect. *Monopodiae*, *Kuepferia*, and *Sinogentiana*, with more cases of heterogeneity likely to be detected if more species were studied. In subtribe Gentianinae, boundary shifts (LSC‐IR and SSC‐IR) had high phylogenetic signal, which is not commonly observed in plants (e.g., Yao et al., 2019; Ye et al., 2018).

It is well known that the early stage of the establishment of an organelle (such as the chloroplast) is characterized by massive gene losses and functional transfers to the nuclear genome (Kleine et al., 2009; Martin et al., 1998), with further gene losses and plastome size reduction as selection pressures change. In the case of the *ndh* complex, some nonfunctional *ndh* gene fragments have been found in the mitochondrial or nuclear genomes of plants experiencing *ndh* losses in the plastome (Daniell et al., 2016; Lin et al., 2015). Also, in orchids, it appears that loss of *ndh* within the chloroplast and nuclear genomes occurred concomitantly (Lin et al., 2017). Nuclear genomic data would be useful to investigate whether the genes absent from the plastid have been integrated into the nucleus or have simply been lost. Being similar to *ndh* genes, *rps16* is another commonly lost plastid gene (Mohanta et al., 2020). In the case of *rps16*, we argue that after its pseudogenization at the base of Gentianinae, its complete loss in some lineages (which are mostly those variable in plastome structure) should be considered as a simple loss. In our study, plastome sizes among all 59 Gentianinae species differed by ~34 kB, with the smallest plastomes less than 120 kB in size, making them substantially smaller than the mean plastid size of 153 kB for land plants (Weng et al., 2017).

### 4.3 Plastome degradation in response to evolutionary rates and ecology

It is generally accepted that plastome degradation and the acceleration of substitution rates can be both caused by shifts in ecologically relevant traits, such as holoparasitism (Wicke et al., 2016) or growing habits (e.g., herbaceous or woody; Schwarz et al., 2017). In subtribe Gentianinae (species that are all herbaceous and photosynthetic) though, elevated substitution rates may be more associated with plastome size and life‐history strategy (e.g., annual vs. perennial), as observed in other plant groups (Gaut et al., 2011; Schwarz et al., 2017). For example, substitution rates show a significant negative correlation with plastome size, although the correlation is not as strong as that seen in legumes (dN, *p* < 0.05; dS, *p* = 0.063; Schwarz et al., 2017). In Gentianinae, the clade with the most extensive gene losses (sect. *Chondrophyllae s. l*.) has the smallest plastomes and a short generation time (i.e., they are annuals). This does not always hold true in subtribe Gentianinae since sect. *Microsperma*, which is also an annual clade, had similar plastome sizes and substitution rates as most perennial groups. Variation is also observed within a clade, for example in *Sinogentiana*, in which only one of the two annual species shows a shift in plastome size and substitution rates (average dN, 0.010; average dS, 0.013), while some perennial clades with smaller plastomes had either elevated substitution rates (monotypic sect. *Phyllocalyx*, average dN, 0.007; average dS, 0.030) or no rate shift (sect. *Kudoa*; average dN, 0; average dS, 0.001).

Most models of plastome evolution show that an increase in microstructural changes and the acceleration of dN and dS may correspond to the first phase of the relaxation of selection (Wicke et al., 2016). In subtribe Gentianinae, some lineages do match this expectation with clades with microstructural changes (*Kuepferia*, sects. *Chondrophyllae s. l*. and *Phyllocalyx*) experiencing an elevated evolutionary rate. Also, for clades characterized by boundary shifts, the main losses tend to be *ndh* genes. However, one exception was detected in sect. *Kudoa*, a clade with microstructural changes, yet without a shift in evolutionary rate. Lineage‐specific rate heterogeneity has been detected in *Pelargonium*, which have highly elevated rather than decelerated dS associated with IR expansion (Weng et al., 2017; Zhu et al., 2016). The occurrence of lineage‐specific rate heterogeneity, either with or without associated microstructural changes, suggests that plastome degradation in subtribe Gentianinae may be more complex than that predicted by widely accepted models of plastome evolution.

### 4.4 Plastome degradation in response to past and present environmental pressures

It is easy to understand why the *ndh* complex could be functionally lost from the plastomes of nonphotosynthetic parasites (Delannoy et al., 2011; Graham et al., 2017; Wicke et al., 2013, 2016); however, it has also been lost in a number of photosynthetic plant lineages (e.g., Braukmann et al., 2009; Graham et al., 2017; Ross et al., 2016; Ruhlman et al., 2015; Ruhlman & Jansen, 2018; Song et al., 2017; Yao et al., 2019). Loss of plastid *ndh* may be explained by genomic redundancy, as *ndh* shares the same function as the independent nuclear PGR5‐dependent pathway (Ruhlman et al., 2015). The *ndh* complex is essential for electron cycling around photosystem I under heat‐stressed conditions, but is less important under cold‐stressed conditions (Wang et al., 2006). In addition, the loss of *ndh* genes in the plastome is also assumed to be related to dry and light‐intensive habitats in *Selaginella* (Xu et al., 2018; Zhang et al., 2019), or similar habitats in *Kingdonia* (Sun et al., 2020). In contrast, it remains challenging to identify an associated habitat characteristic explaining gene losses in *Gentiana*. In this genus, the loss of the *ndh* complex was only observed in some lineages occurring in the QTP, and not in those only or mainly from Europe (*G*. sect. *Calathianae*, *G*. sect. *Ciminalis*, and *G*. sect. *Gentiana*) or North America (*G*. sect. *Pneumonanthe*). Yet, species of *Gentiana* in the QTP do not occur in particularly dry habitats. For example, gene loss was detected in *G. phyllocalyx*, which grows in lush alpine meadows experiencing a wet summer climate. The habitats of sects. *Chondrophyllae s. l*., *Monopodiae,* and *Kudoa*, although variable, are also not usually characterized by severe droughts. Even if some species do occur in drier environments, such as *G. dahurica* (sect. *Cruciata*) which may occur around dunes, no gene losses were found in this species. Finally, *ndh* losses were not detected in cold‐tolerant lineages such as sect. *Frigida* and sect. *Monopodiae* I, of which many species can occur at very high elevations.

Nevertheless, gene losses occurred in the past when species may have experienced different environmental conditions. Indeed, the lineage‐specific patterns of microstructural change that we found across subtribe Gentianinae make this group attractive for further investigations as to whether plastid microstructural changes are associated with historical climate or geological change. Despite the uncertainty associated with the use of secondary calibration points in dated phylogenetic analyses (Schenk & Axel, 2016), we recover node ages of Gentianeae, Gentianinae, and *Gentiana*, which are similar to earlier studies with different calibration schemes (e.g., Favre et al., 2016). *Gentiana* originated, and is currently most species‐rich, in the region of the QTP that has experienced a dynamic climatic and geological history (reviewed, for example, in Favre et al., 2015). However, divergence dating showed that independent *ndh* gene losses in different Gentianinae lineages occurred through the Eocene, Miocene, and Pliocene, suggesting heterogenous triggers of *ndh* losses. For instance, the *ndh* losses in sect. *Chondrophyllae s. l*. were likely to have occurred between the Eocene and Oligocene, relatively early in the evolution of the alpine flora of the region (Ding et al., 2020). During this time, the uplift of the QTP was progressing (Favre et al., 2015; Mulch & Chamberlain, 2005), creating new habitats that may have had sparse vegetation. Gene losses in sect. *Kudoa* were more recent, occurring in the Miocene. This was a time of major orogenic change where there was major mountain uplift (Favre et al., 2015). The uplift of the Himalayas in particular cast a rain shadow that contributed to progressive aridification of the plateau platform and resulted in more pronounced seasonality of precipitation (Ding et al., 2020; Favre et al., 2015). Therefore, lower precipitation (or stronger seasonality) may have favored the lineage of sect. *Kudoa*. Today, some species of this section also occur in relatively wet habitats, but this habitat shift may have occurred more recently (after gene loss), during the diversification of the section, as habitat preferences diversified more recently. However, considerable uncertainty remains in the timing of historical climate and geologic changes in the QTP (see review in Favre et al., 2015), and more direct evidence is needed to understand the drivers of plastome structural changes in Gentianinae.

### 4.5 Conclusion

By sampling all main lineages in subtribe Gentianinae, we have discovered diverse patterns of plastid genome degradation that have resulted in considerable variation in plastome size, sometimes due to particularly short single‐copy regions or the loss of functional genes. Repeated gene losses occurred predominantly in annuals, as well as some perennials such as sections *Kudoa* and *Phyllocalyx*. Microstructural change in the plastome was generally very similar among species belonging to one section or genus, except in some sections such as sect. *Monopodiae*, which had more complex patterns than expected. In addition, elevated evolutionary rates were usually detected in taxa with microstructural changes (e.g., gene losses and boundary shifts), with the exception of sect. *Kudoa*. Our study thus suggests that the different lineages of subtribe Gentianinae have experienced contrasting evolutionary pressures.

## CONFLICT OF INTEREST

None declared.

## AUTHOR CONTRIBUTIONS

**Peng‐Cheng Fu:** Conceptualization (equal); data curation (equal); formal analysis (equal); investigation (equal); supervision (equal); writing – original draft (equal); writing – review and editing (equal). **Shan‐Shan Sun:** Formal analysis (equal); visualization (equal); writing – original draft (supporting). **Alex D. Twyford:** Formal analysis (equal); writing – review and editing (equal). **Bei‐Bei Li:** Formal analysis (equal); investigation (equal). **Rui‐Qi Zhou:** Formal analysis (equal); investigation (equal). **Shi‐Long Chen:** Data curation (equal); writing – review and editing (supporting). **Qing‐Bo Gao:** Data curation (equal); writing – writing – review and editing (supporting). **Adrien Favre:** Conceptualization (equal); data curation (equal); writing – original draft (equal); writing – review and editing (equal).

## Supporting information

Supplementary Material

Supplementary Material

Supplementary Material

Supplementary Material

Supplementary Material

Supplementary Material

## Data Availability

Main data are provided within the text, tables, figures, appendix, and supplementary. Supporting data are uploaded to Dryad (https://doi.org/10.5061/dryad.h70rxwdgw).
