# Supplementary material for: Lineage‐specific plastid degradation in subtribe Gentianinae (Gentianaceae)
Source: Ecol Evol. 2021 Feb 22;11(7):3286–99. doi: 10.1002/ece3.7281 (PMC8019047; doi:10.1002/ece3.7281)
Supplement: Supplementary file 1 — Supplementary Material [file ECE3-11-3286-s003.docx]

**TABLE A2** List of primers used for verifying a long insertion in *Gentiana cuneibarba.*

| Name | Sequence (5'-3') |
| --- | --- |
| Gcun1-F2 | ACTCGAACAAATCGAAACTT |
| Gcun1-R2 | ATACTCGCATGAACAACCTT |
| Gcun2-F1 | TTGTCATAAAACATCACCCC |
| Gcun2-R1 | CAGACCTAGCATTTCCTCGC |
| Gcun3-F2 | TACCACTAACTACACTCGCT |
| Gcun3-R2 | GGGTTGACTTATTAATATGC |
