# Supplementary material for: Lineage‐specific plastid degradation in subtribe Gentianinae (Gentianaceae)
Source: Ecol Evol. 2021 Feb 22;11(7):3286–99. doi: 10.1002/ece3.7281 (PMC8019047; doi:10.1002/ece3.7281)
Supplement: Supplementary file 4 — Supplementary Material [file ECE3-11-3286-s006.pdf]

# SUPPLEMENTARY B

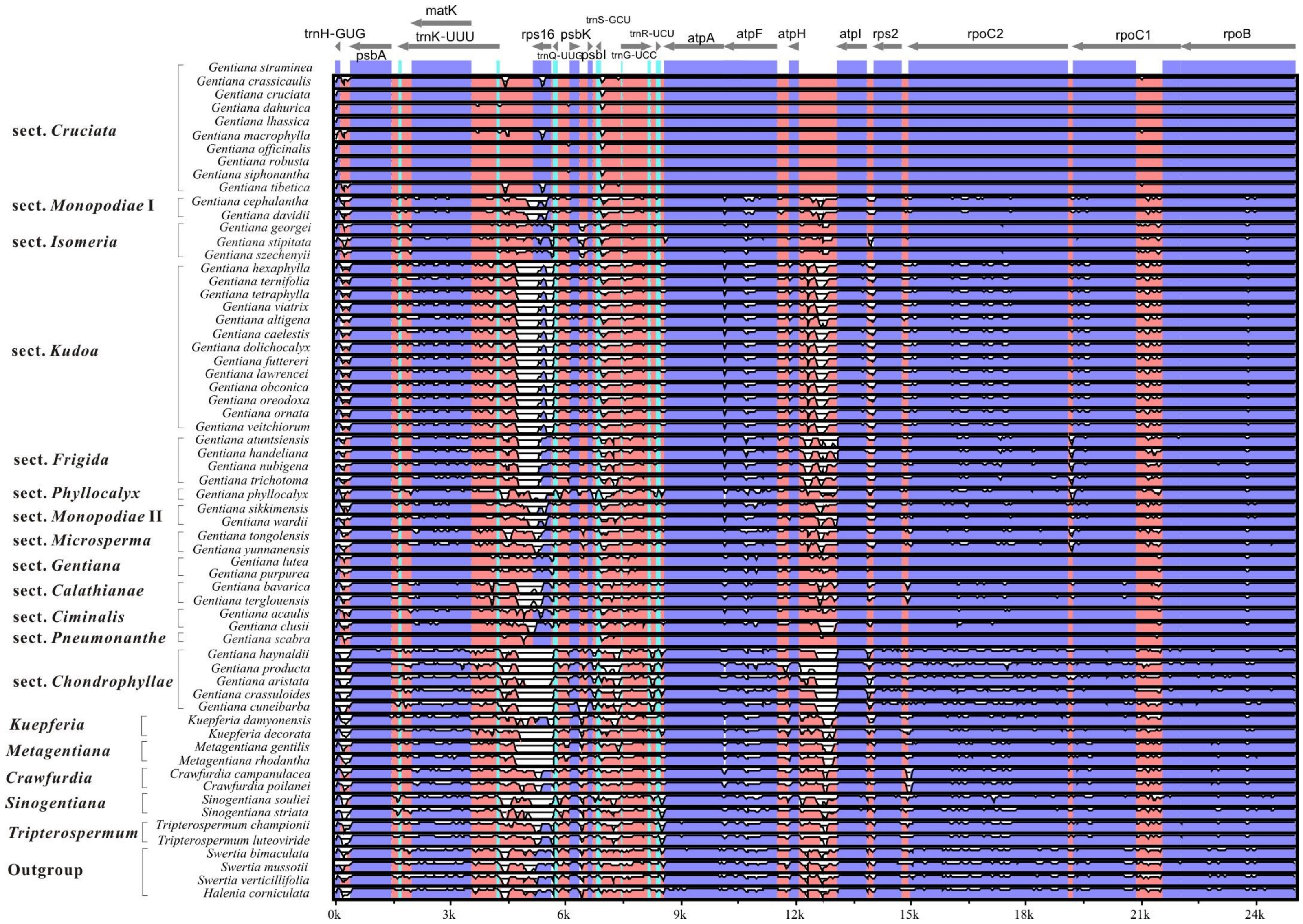

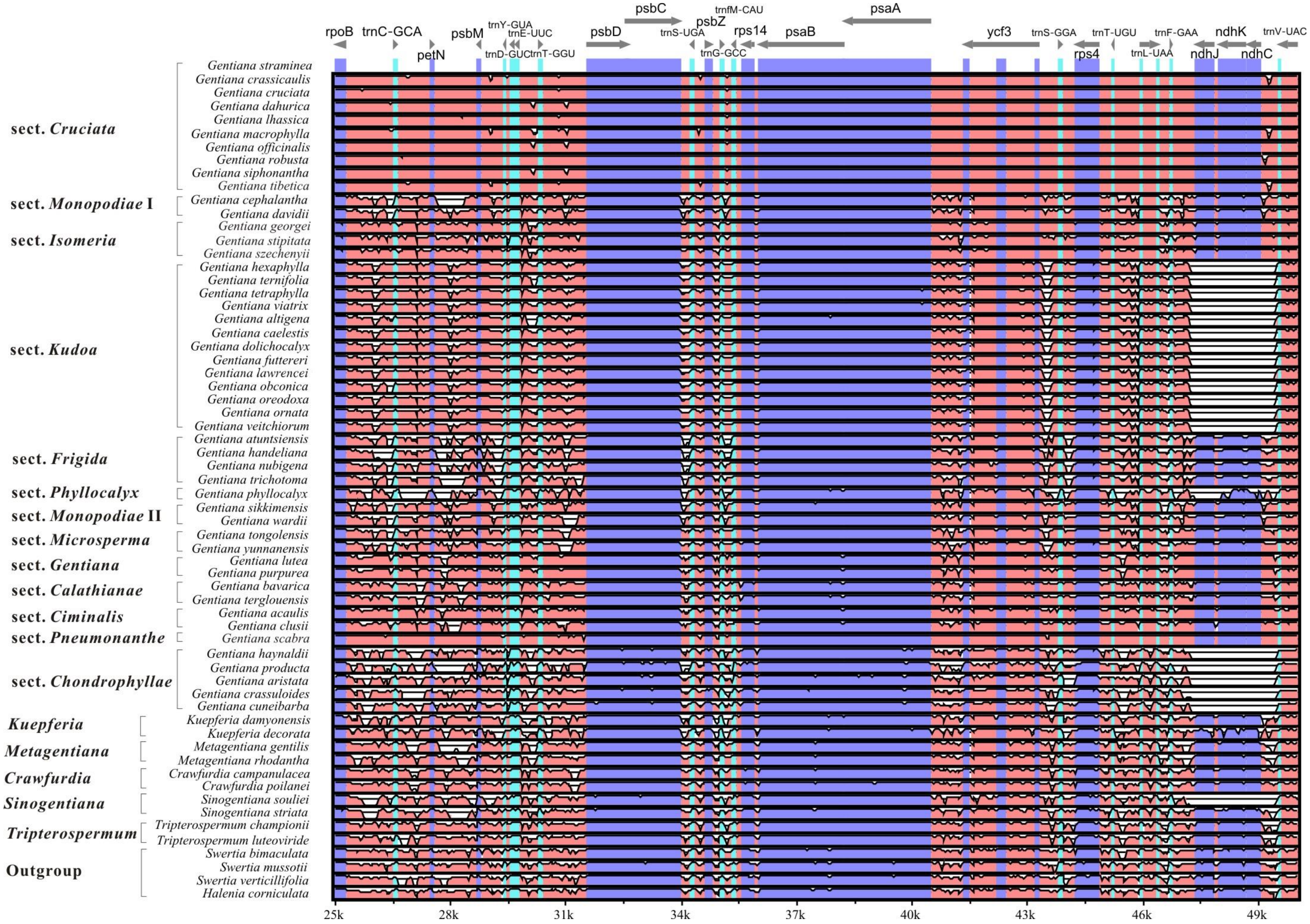

rpoB
trnC-GCA
petN
psbM
trnY-GUA
trnE-UUC
trnD-GUC
trnT-GGU
psbD
psbC
trnS-UGA
psbZ
trnfM-CAU
trnG-GCC
rps14
psaB
psaA
ycf3
trnS-GGA
rps4
trnT-UGU
trnL-UAA
trnF-GAA
ndhJ
ndhK
ndhC
trnV-UAC
sect. Cruciata
Gentiana straminea
Gentiana crassicaulis
Gentiana cruciata
Gentiana dahurica
Gentiana lhassica
Gentiana macrophylla
Gentiana officinalis
Gentiana robusta
Gentiana siphonantha
Gentiana tibetica
sect. Monopodiae I
Gentiana cephalantha
Gentiana davidii
sect. Isomeria
Gentiana georgei
Gentiana stipitata
Gentiana szechenyii
sect. Kudoa
Gentiana hexaphylla
Gentiana ternifolia
Gentiana tetraphylla
Gentiana viatrix
Gentiana altigena
Gentiana caelestis
Gentiana dolichocalyx
Gentiana futtereri
Gentiana lawrencei
Gentiana obconica
Gentiana oreodoxa
Gentiana ornata
Gentiana veitchiorum
sect. Frigida
Gentiana atuntsiensis
Gentiana handeliana
Gentiana nubigena
Gentiana trichotoma
sect. Phyllocalyx
Gentiana phyllocalyx
sect. Monopodiae II
Gentiana sikkimensis
Gentiana wardii
sect. Microsperma
Gentiana tongolensis
Gentiana yunnanensis
sect. Gentiana
Gentiana lutea
Gentiana purpurea
sect. Calathianae
Gentiana bavarica
Gentiana terglouensis
sect. Ciminalis
Gentiana acaulis
Gentiana clusii
sect. Pneumonanthe
Gentiana scabra
sect. Chondrophyllae
Gentiana haynaldii
Gentiana producta
Gentiana aristata
Gentiana crassuloides
Gentiana cuneibarba
Kuepferia
Kuepferia damyonensis
Kuepferia decorata
Metagentiana
Metagentiana gentilis
Metagentiana rhodantha
Crawfurdia
Crawfurdia campanulacea
Crawfurdia poilanei
Sinogentiana
Sinogentiana souliei
Sinogentiana striata
Tripterospermum
Tripterospermum championii
Tripterospermum luteoviride
Outgroup
Swertia bimaculata
Swertia mussotii
Swertia verticillifolia
Halenia corniculata
25k
28k
31k
34k
37k
40k
43k
46k
49k

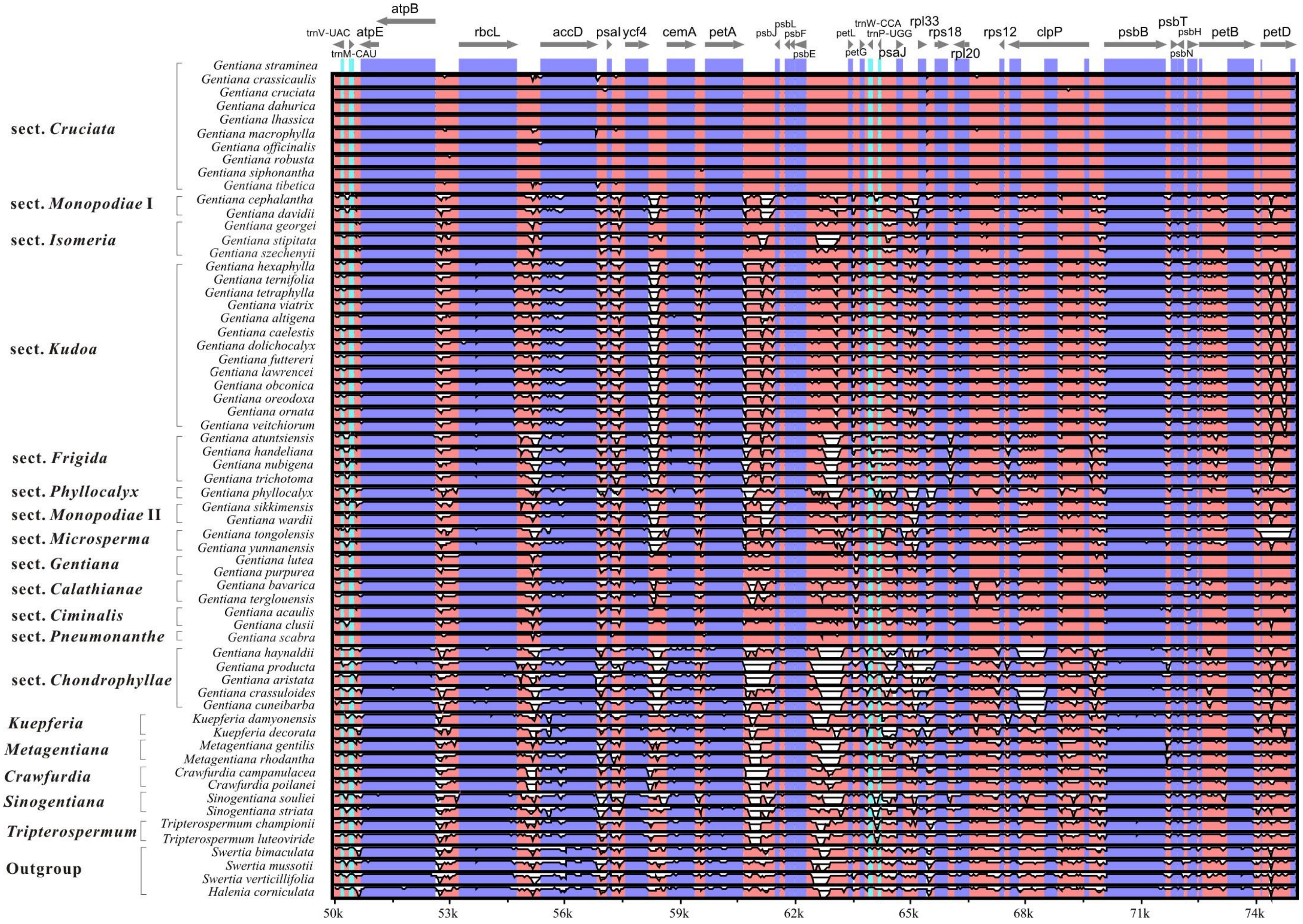
atpB
trnV-UAC
atpE
trnM-CAU
rbcL
accD
psaI
ycf4
cemA
petA
psbJ
psbL
psbF
psbE
petL
petG
trnW-CCA
trnP-UGG
psaJ
rpl33
rps18
rpl20
rps12
clpP
psbB
psbT
psbH
psbN
petB
petD
sect. Cruciata
Gentiana straminea
Gentiana crassicaulis
Gentiana cruciata
Gentiana dahurica
Gentiana lhassica
Gentiana macrophylla
Gentiana officinalis
Gentiana robusta
Gentiana siphonantha
Gentiana tibetica
sect. Monopodiae I
Gentiana cephalantha
Gentiana davidii
sect. Isomeria
Gentiana georgei
Gentiana stipitata
Gentiana szechenyii
sect. Kudoa
Gentiana hexaphylla
Gentiana ternifolia
Gentiana tetraphylla
Gentiana viatrix
Gentiana altigena
Gentiana caelestis
Gentiana dolichocalyx
Gentiana futtereri
Gentiana lawrencei
Gentiana obconica
Gentiana oreodoxa
Gentiana ornata
Gentiana veitchiorum
sect. Frigida
Gentiana atuntsiensis
Gentiana handeliana
Gentiana nubigena
Gentiana trichotoma
sect. Phyllocalyx
Gentiana phyllocalyx
sect. Monopodiae II
Gentiana sikkimensis
Gentiana wardii
sect. Microsperma
Gentiana tongolensis
Gentiana yunnanensis
sect. Gentiana
Gentiana lutea
Gentiana purpurea
sect. Calathianae
Gentiana bavarica
Gentiana terglouensis
sect. Ciminalis
Gentiana acaulis
Gentiana clusii
sect. Pneumonanthe
Gentiana scabra
sect. Chondrophyllae
Gentiana haynaldii
Gentiana producta
Gentiana aristata
Gentiana crassuloides
Gentiana cuneibarba
Kuepferia
Kuepferia damyonensis
Kuepferia decorata
Metagentiana
Metagentiana gentilis
Metagentiana rhodantha
Crawfurdia
Crawfurdia campanulacea
Crawfurdia poilanei
Sinogentiana
Sinogentiana souliei
Sinogentiana striata
Tripterospermum
Tripterospermum championii
Tripterospermum luteoviride
Outgroup
Swertia bimaculata
Swertia mussotii
Swertia verticillifolia
Halenia corniculata
50k
53k
56k
59k
62k
65k
68k
71k
74k

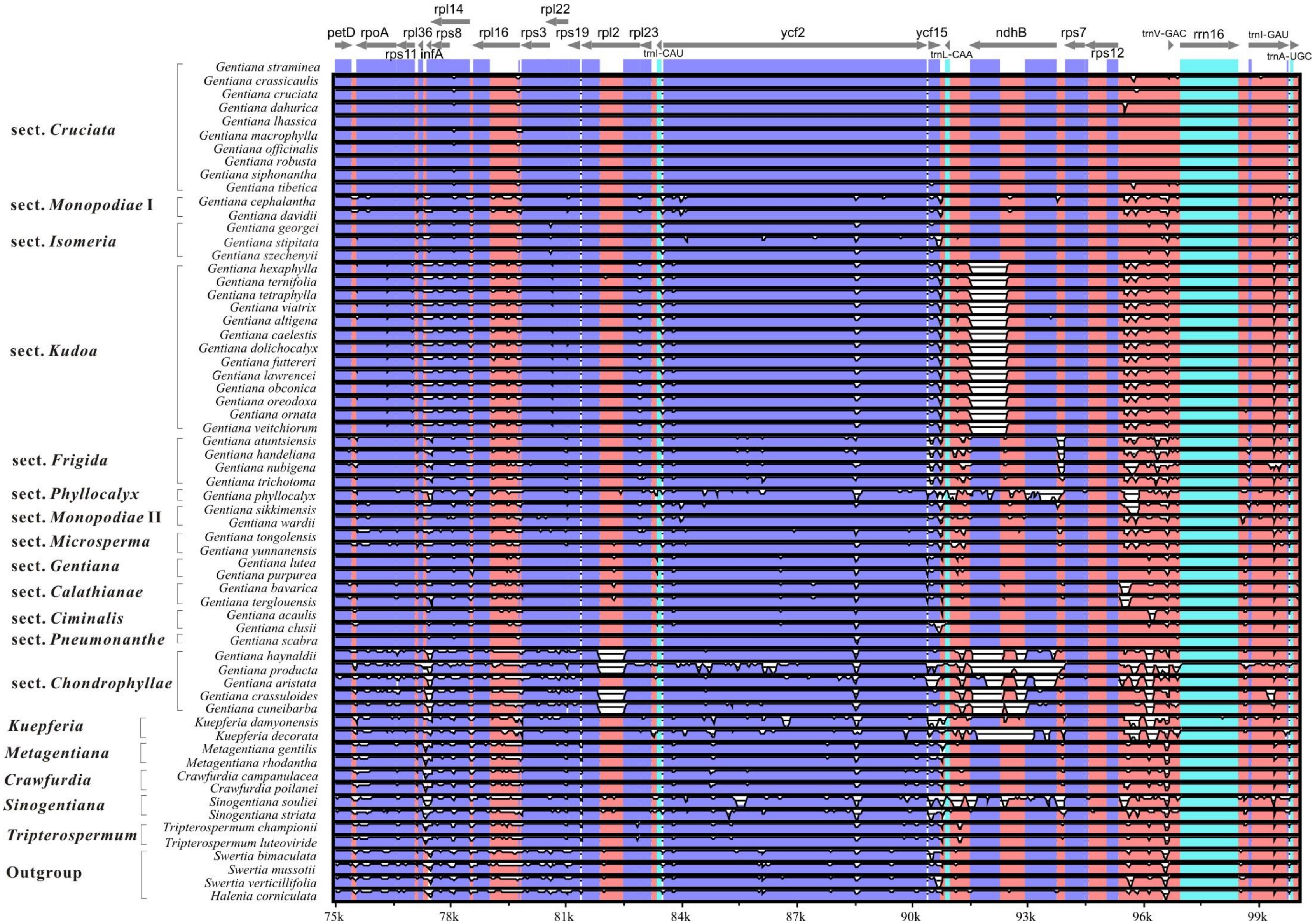
petD
rpoA
rps11
rpl14
rpl36
infA
rps8
rpl16
rps3
rpl22
rps19
rpl2
rpl23
trnI-CAU
ycf2
ycf15
trnL-CAA
ndhB
rps7
rps12
trnV-GAC
rrn16
trnI-GAU
trnA-UGC
sect. Cruciata
Gentiana straminea
Gentiana crassicaulis
Gentiana cruciata
Gentiana dahurica
Gentiana lhassica
Gentiana macrophylla
Gentiana officinalis
Gentiana robusta
Gentiana siphonantha
Gentiana tibetica
sect. Monopodiae I
Gentiana cephalantha
Gentiana davidii
sect. Isomeria
Gentiana georgei
Gentiana stipitata
Gentiana szechenyii
sect. Kudoa
Gentiana hexaphylla
Gentiana ternifolia
Gentiana tetraphylla
Gentiana viatrix
Gentiana altigena
Gentiana caelestis
Gentiana dolichocalyx
Gentiana futtereri
Gentiana lawrencei
Gentiana obconica
Gentiana oreodoxa
Gentiana ornata
Gentiana veitchiorum
sect. Frigida
Gentiana atuntsiensis
Gentiana handeliana
Gentiana nubigena
Gentiana trichotoma
sect. Phyllocalyx
Gentiana phyllocalyx
sect. Monopodiae II
Gentiana sikkimensis
Gentiana wardii
sect. Microsperma
Gentiana tongolensis
Gentiana yunnanensis
sect. Gentiana
Gentiana lutea
Gentiana purpurea
sect. Calathianae
Gentiana bavarica
Gentiana terglouensis
sect. Ciminalis
Gentiana acaulis
Gentiana clusii
sect. Pneumonanthe
Gentiana scabra
sect. Chondrophyllae
Gentiana haynaldii
Gentiana producta
Gentiana aristata
Gentiana crassuloides
Gentiana cuneibarba
Kuepferia
Kuepferia damyonensis
Kuepferia decorata
Metagentiana
Metagentiana gentilis
Metagentiana rhodantha
Crawfurdia
Crawfurdia campanulacea
Crawfurdia poilanei
Sinogentiana
Sinogentiana souliei
Sinogentiana striata
Tripterospermum
Tripterospermum championii
Tripterospermum luteoviride
Outgroup
Swertia bimaculata
Swertia mussotii
Swertia verticillifolia
Halenia corniculata
75k
78k
81k
84k
87k
90k
93k
96k
99k

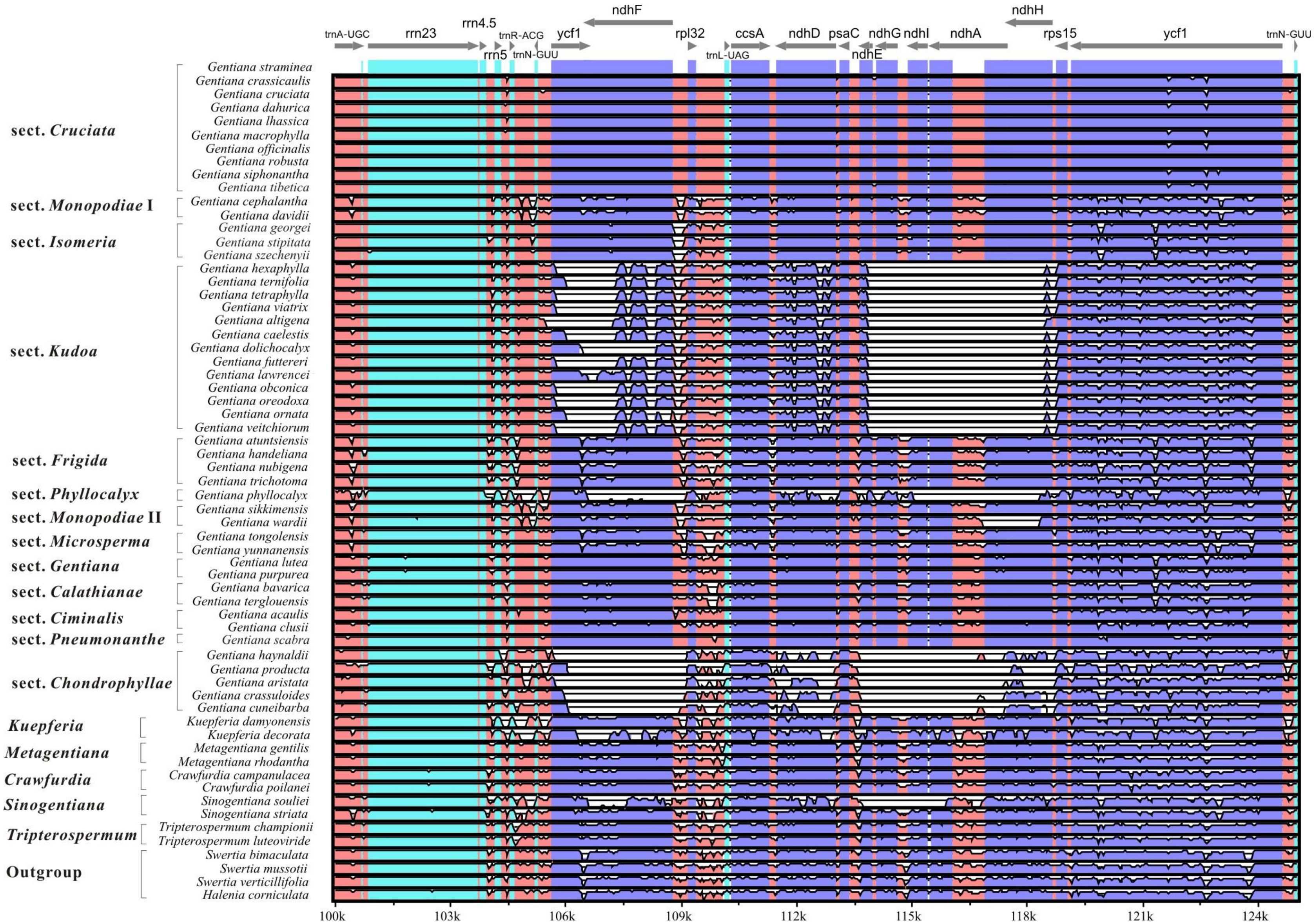
ndhF
ndhH
rrn4.5
trnA-UGC
rrn23
trnR-ACG
ycf1
rpl32
ccsA
ndhD
psaC
ndhG
ndhI
ndhA
rps15
ycf1
trnN-GUU
rrn5
trnN-GUU
trnL-UAG
ndhE
sect. Cruciata
Gentiana straminea
Gentiana crassicaulis
Gentiana cruciata
Gentiana dahurica
Gentiana lhassica
Gentiana macrophylla
Gentiana officinalis
Gentiana robusta
Gentiana siphonantha
Gentiana tibetica
sect. Monopodiae I
Gentiana cephalantha
Gentiana davidii
sect. Isomeria
Gentiana georgei
Gentiana stipitata
Gentiana szechenyii
sect. Kudoa
Gentiana hexaphylla
Gentiana ternifolia
Gentiana tetraphylla
Gentiana viatrix
Gentiana altigena
Gentiana caelestis
Gentiana dolichocalyx
Gentiana futtereri
Gentiana lawrencei
Gentiana obconica
Gentiana oreodoxa
Gentiana ornata
Gentiana veitchiorum
sect. Frigida
Gentiana atuntsiensis
Gentiana handeliana
Gentiana nubigena
Gentiana trichotoma
sect. Phyllocalyx
Gentiana phyllocalyx
sect. Monopodiae II
Gentiana sikkimensis
Gentiana wardii
sect. Microsperma
Gentiana tongolensis
Gentiana yunnanensis
sect. Gentiana
Gentiana lutea
Gentiana purpurea
sect. Calathianae
Gentiana bavarica
Gentiana terglouensis
sect. Ciminalis
Gentiana acaulis
Gentiana clusii
sect. Pneumonanthe
Gentiana scabra
sect. Chondrophyllae
Gentiana haynaldii
Gentiana producta
Gentiana aristata
Gentiana crassuloides
Gentiana cuneibarba
Kuepferia
Kuepferia damyonensis
Kuepferia decorata
Metagentiana
Metagentiana gentilis
Metagentiana rhodantha
Crawfurdia
Crawfurdia campanulacea
Crawfurdia poilanei
Sinogentiana
Sinogentiana souliei
Sinogentiana striata
Tripterospermum
Tripterospermum championii
Tripterospermum luteoviride
Outgroup
Swertia bimaculata
Swertia mussotii
Swertia verticillifolia
Halenia corniculata
100k
103k
106k
109k
112k
115k
118k
121k
124k

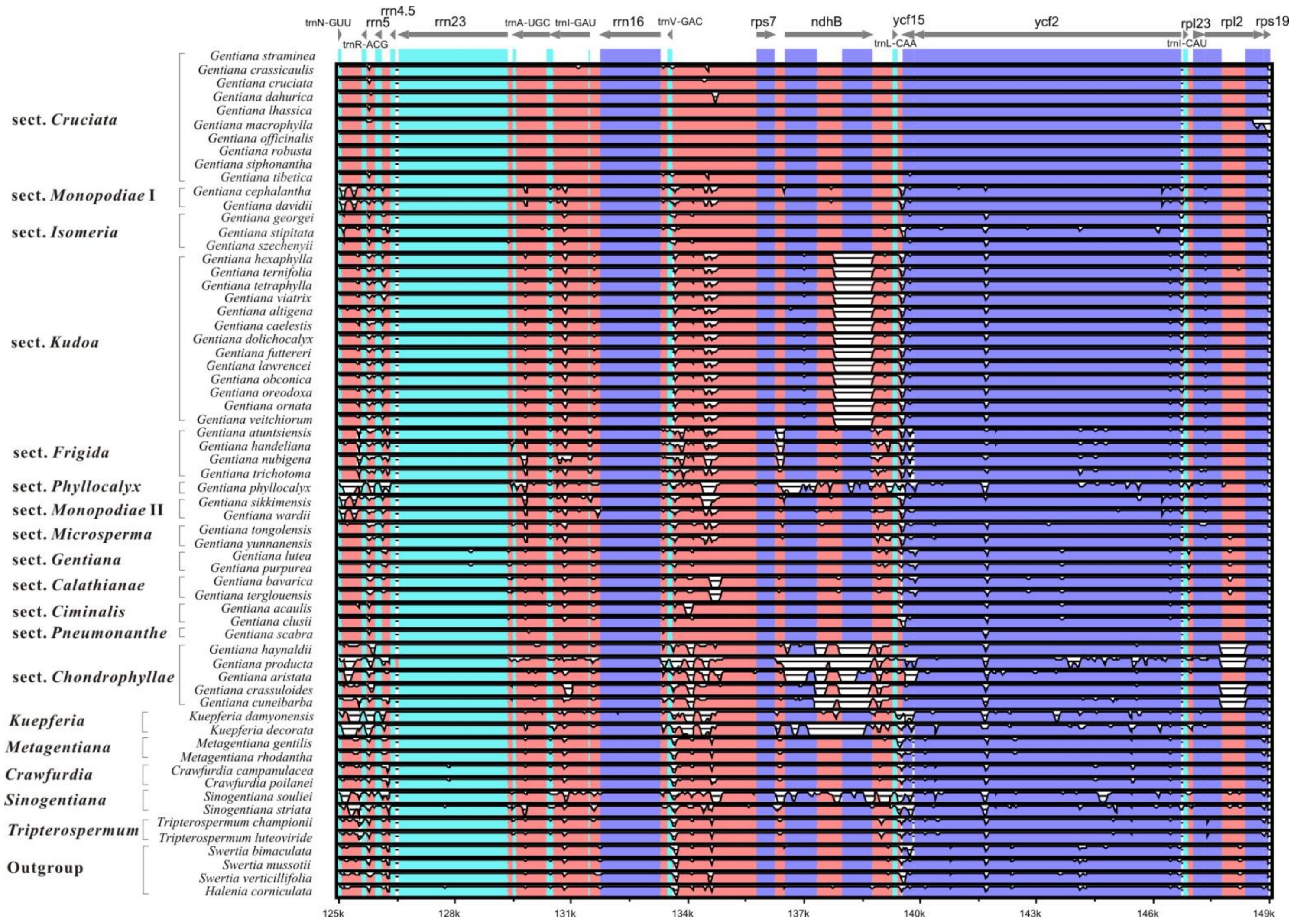


**FIGURE B1** Plastome comparison in subtribe Gentianeae using *Gentiana straminea* as the reference. The arrows on the top show the direction of genes.
