# Supplementary material for: Lineage‐specific plastid degradation in subtribe Gentianinae (Gentianaceae)
Source: Ecol Evol. 2021 Feb 22;11(7):3286–99. doi: 10.1002/ece3.7281 (PMC8019047; doi:10.1002/ece3.7281)
Supplement: Supplementary file 5 — Supplementary Material [file ECE3-11-3286-s002.pdf]

APPENDIX B

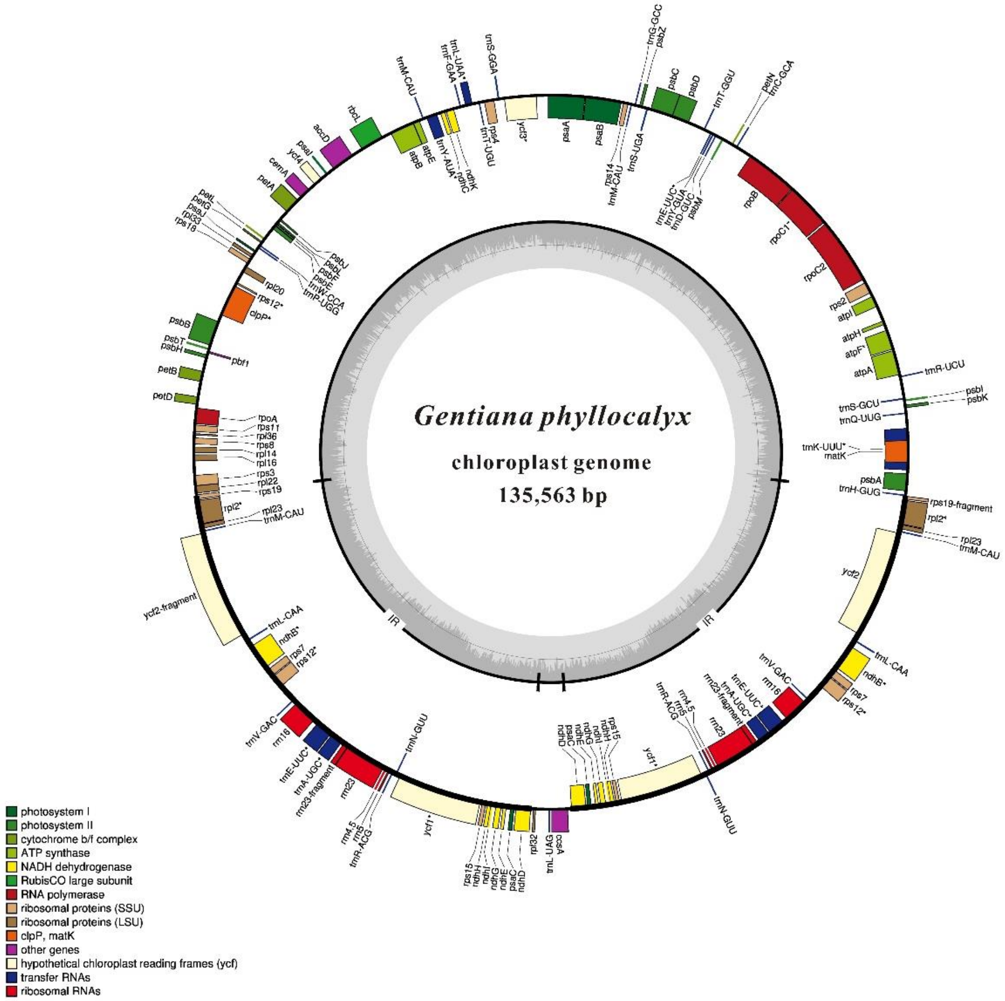

FIGURE B1 Map of the plastome of *Gentiana phyllocalyx*. Genes drawn inside the circle are transcribed clockwise, and those drawn outside are transcribed counterclockwise. Genes belonging to different functional groups are shown in different colours.

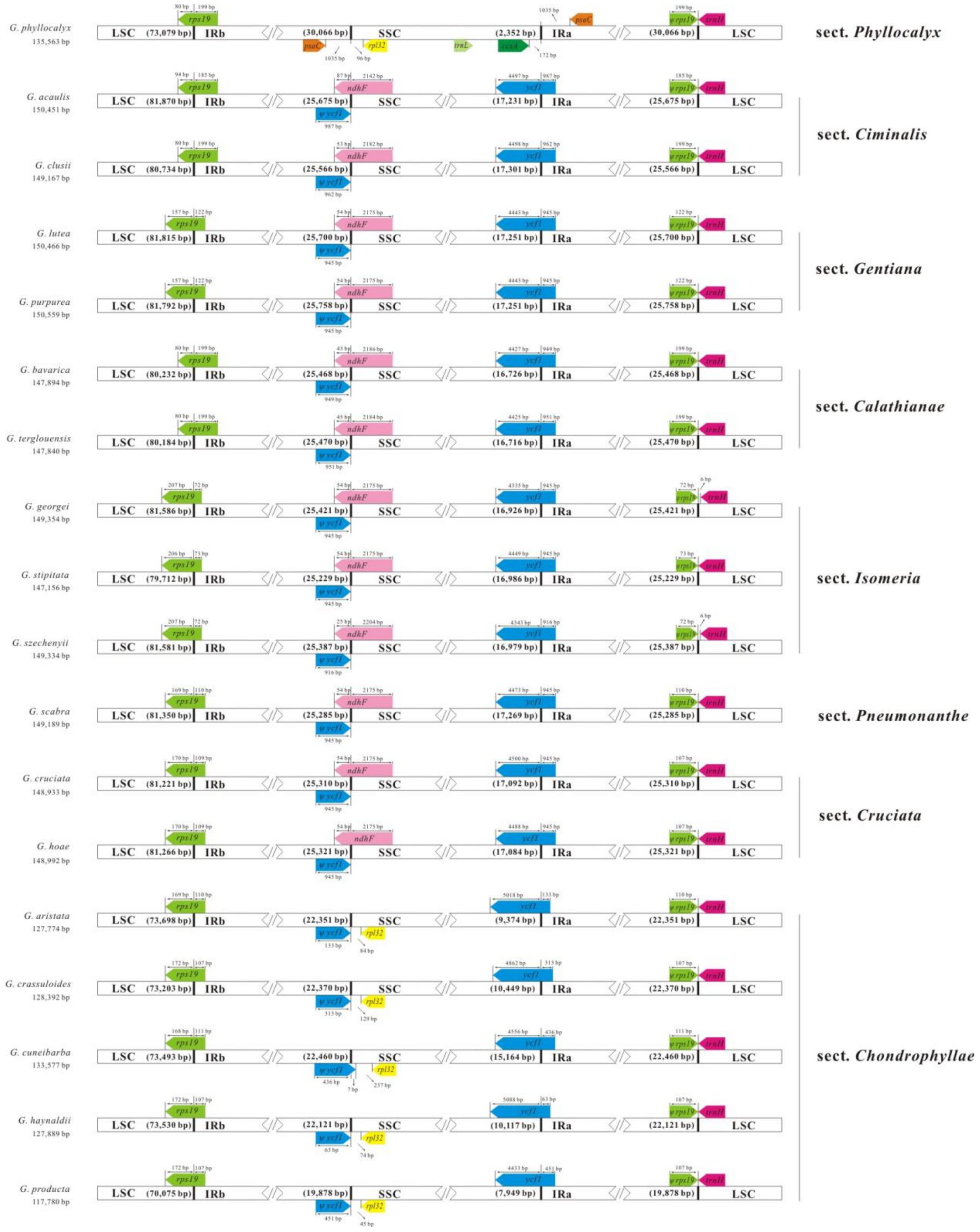

G. phyllocalyx 135,563 bp
G. acaulis 150,451 bp
G. clusii 149,167 bp
G. lutea 150,466 bp
G. purpurea 150,559 bp
G. bavarica 147,894 bp
G. terglouensis 147,840 bp
G. georgei 149,354 bp
G. stipitata 147,156 bp
G. szechenyii 149,334 bp
G. scabra 149,189 bp
G. cruciata 148,933 bp
G. hoae 148,992 bp
G. aristata 127,774 bp
G. crassuloides 128,392 bp
G. cuneibarba 133,577 bp
G. haynaldii 127,889 bp
G. producta 117,780 bp
sect. Phyllocalyx
sect. Ciminalis
sect. Gentiana
sect. Calathianae
sect. Isomeria
sect. Pneumonanthe
sect. Cruciata
sect. Chondrophyllae

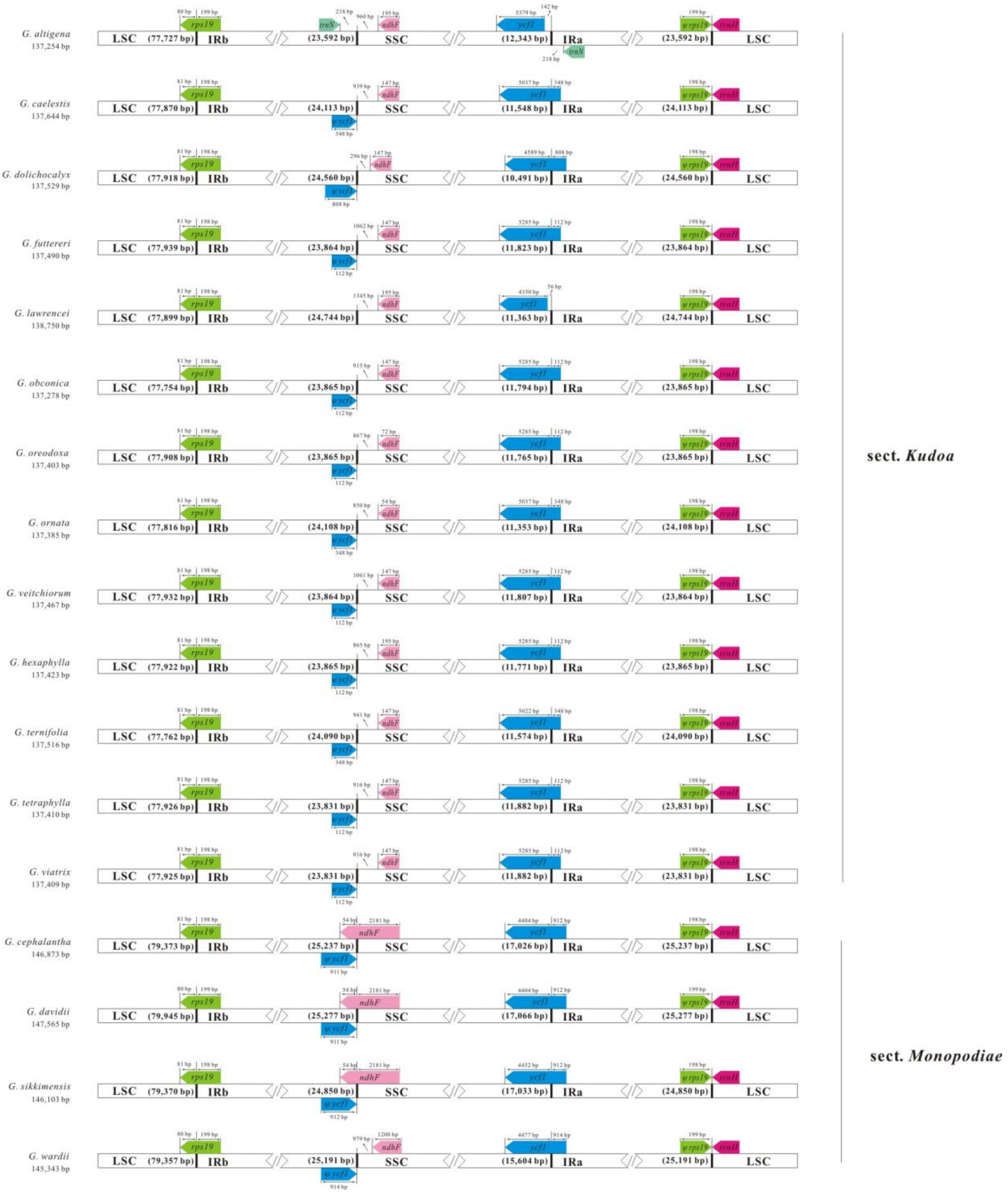

G. altigena
137,254 bp
G. caelestis
137,644 bp
G. dolichocalyx
137,529 bp
G. futtereri
137,490 bp
G. lawrencei
138,750 bp
G. obconica
137,278 bp
G. oreodoxa
137,403 bp
G. ornata
137,385 bp
G. veitchiorum
137,467 bp
G. hexaphylla
137,423 bp
G. ternifolia
137,516 bp
G. tetraphylla
137,410 bp
G. viatrix
137,409 bp
G. cephalantha
146,873 bp
G. davidii
147,565 bp
G. sikkimensis
146,103 bp
G. wardii
145,343 bp
LSC
IRb
SSC
IRa
sect. Kudoa
sect. Monopodiae

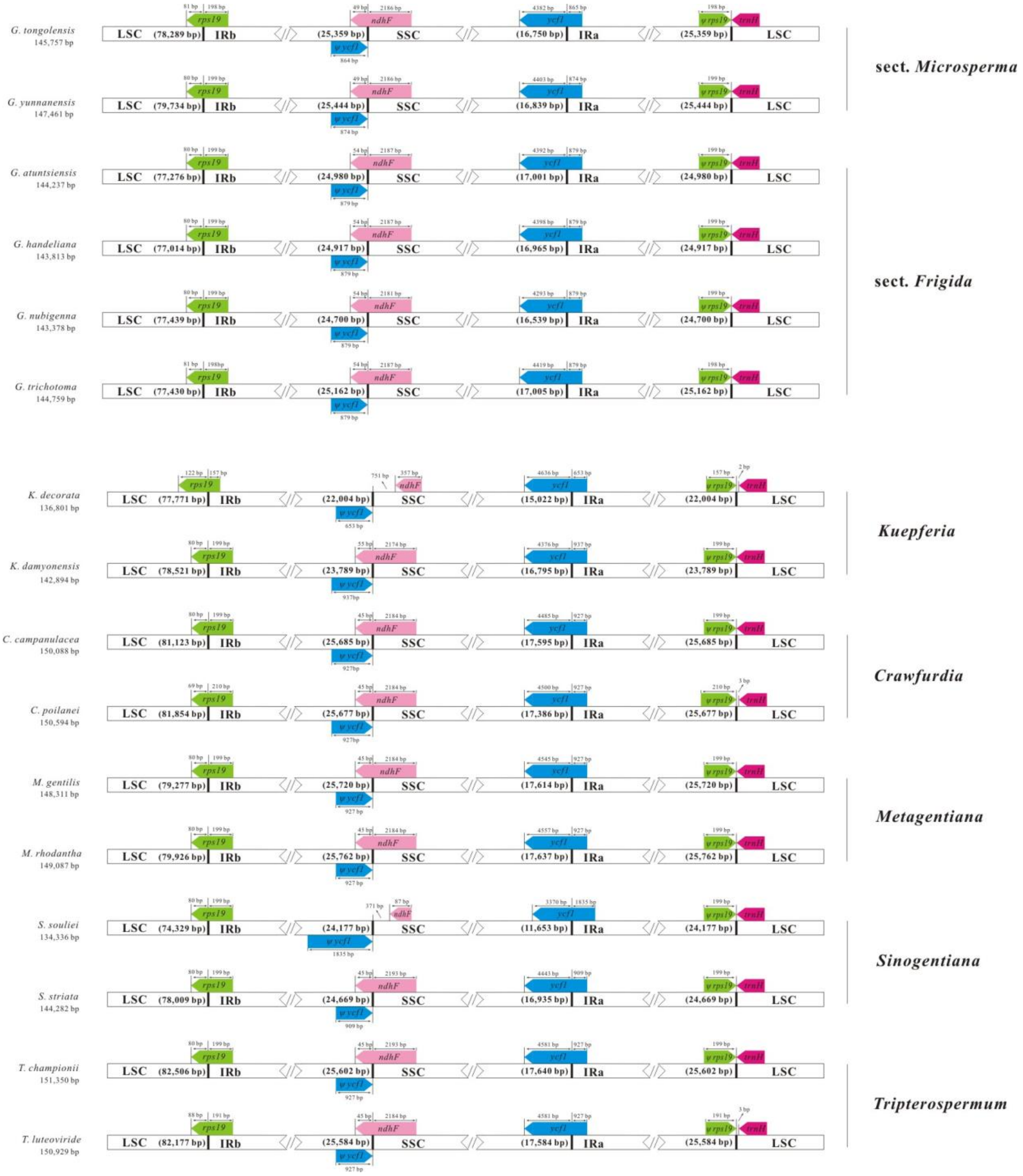

FIGURE B2 Comparison of LSC, IRs, and SSC junction positions among all newly sequenced plastomes in subtribe Gentianeae, along with seven published plastomes in *Gentiana* sect. *Kudoa*.
