# Supplementary material for: Lineage‐specific plastid degradation in subtribe Gentianinae (Gentianaceae)
Source: Ecol Evol. 2021 Feb 22;11(7):3286–99. doi: 10.1002/ece3.7281 (PMC8019047; doi:10.1002/ece3.7281)
Supplement: Supplementary file 6 — Supplementary Material [file ECE3-11-3286-s004.docx]

**APPENDIX A**

**TABLE A1** Herbarium voucher information for Gentianeae species newly sequenced. The herbarium of Luoyang Normal University has no acronym at present and is abbreviated with HLY.

| Genus | Species | Author | Collection No. | Location | Country | Deposition location |
| --- | --- | --- | --- | --- | --- | --- |
| *Gentiana* | *acaulis* | L. | AFIT-18001 | Grand-Saint Bernard, Aosta | Italy | FR |
| *Gentiana* | *altigena* | H. Smith | Fu2018079-12 | Gongshan, Yunnan | China | HLY |
| *Gentiana* | *aristata* | Maximowicz | Fu2017031-3 | Chenduo, Qinghai | China | HLY |
| *Gentiana* | *atuntsiensis* | W. W. Smith | Fu2018039-1 | Xianggelila, Yunnan | China | HLY |
| *Gentiana* | *bavarica* | L. | AFCH-18016 | La Seya, Valais | Switzerland | FR |
| *Gentiana* | *cephalantha* | Franchet | Fu2016123-4 | Lijiang, Yunnan | China | HLY |
| *Gentiana* | *clusii* | Perrier & Songeon | AFAT-18006a | Sanntaler Sattel, Carinthia | Austria | FR |
| *Gentiana* | *crassuloides* | Bureau & Franchet | Fu2016031-10 | Lixian, Sichuan | China | HLY |
| *Gentiana* | *cruciata* | L. | AF-CH-18005 | Above Fully, Valais | Switzerland | FR |
| *Gentiana* | *cuneibarba* | H. Smith | Fu2018089-1 | Chayu, Tibet | China | HLY |
| *Gentiana* | *davidii* | Franchet | Fu2018220-1 | Zherong, Fujian | China | HLY |
| *Gentiana* | *dolichocalyx* | T. N. Ho | Fu2017204-1 | Aba, Sichuan | China | HLY |
| *Gentiana* | *futtereri* | Diels & Gilg | Fu2017263-2 | Gande, Qinghai | China | HLY |
| *Gentiana* | *georgei* | Diels | Fu2018137-8 | Chayu, Tibet | China | HLY |
| *Gentiana* | *handeliana* | H. Smith | Fu2018087-1 | Chayu, Tibet | China | HLY |
| *Gentiana* | *haynaldii* | Kanitz | Fu2018166-1 | Leiwuqi, Tibet | China | HLY |
| *Gentiana* | *hoae* | P.C. Fu & S.L. Chen | Fu2017072-6 | Nangqian, Qinghai | China | HLY |
| *Gentiana* | *lutea* | L. | AFCH-18001 | Tseuzier Lake, Valais | Switzerland | FR |
| *Gentiana* | *nubigena* | Edgeworth | Fu2017270-6 | Gande, Qinghai | China | HLY |
| *Gentiana* | *phyllocalyx* | C. B. Clarke | Fu2018078 | Gongshan, Yunnan | China | HLY |
| *Gentiana* | *producta* | T. N. Ho | Fu2017242-2 | Jiuzhi, Qinghai | China | HLY |
| *Gentiana* | *purpurea* | L. | AFCH-18004 | Lower Fully Lake, Valais | Switzerland | FR |
| *Gentiana* | *scabra* | Bunge | Fu2018200-6 | Benxi, Liaoning | China | HLY |
| *Gentiana* | *sikkimensis* | C. B. Clarke | Fu2016196-1 | Linzhi, Tibet | China | HLY |
| *Gentiana* | *szechenyii* | Kanitz | Fu2017221-2 | Jiuzhi, Qinghai | China | HLY |
| *Gentiana* | *terglouensis* | Hacquet | AFAT-18007c | Sanntaler Sattel, Carinthia | Austria | FR |
| *Gentiana* | *ternifolia* | Franchet | Fu2017170-8 | Luohuo, Sichuan | China | HLY |
| *Gentiana* | *tetraphylla* | Maximowicz ex Kusnezow | Fu2017171-12 | Luohuo, Sichuan | China | HLY |
| *Gentiana* | *viatrix* | Maximowicz ex Kusnezow | Fu2017172-5 | Luohuo, Sichuan | China | HLY |
| *Gentiana* | *wardii* | W. W. Smith | Fu2018092-7 | Chayu, Tibet | China | HLY |
| *Gentiana* | *yunnanensis* | Franchet | Fu2018023-1 | Lijiang, Yunnan | China | HLY |
| *Kuepferia* | *damyonensis* | (C.Marquand) Adr.Favre | Fu2018101-1 | Chayu, Tibet | China | HLY |
| *Kuepferia* | *decorata* | (Diels) Adr.Favre | Fu2018099-2 | Chayu, Tibet | China | HLY |
| *Metagentiana* | *gentilis* | (Franchet) T.N. Ho & S.W. Liu | AFCN18-244 | Kunming, Yunnan | China | KUN |
| *Metagentiana* | *rhodantha* | (Franchet) T.N. Ho & S.W. Liu | Fu2018210-2 | Dengfeng, Henan | China | HLY |
| *Crawfurdia* | *campanulacea* | Wallich & Griffith ex C. B. Clarke | Favre 214 | Dulongjiang, Yunnan | China | KUN |
| *Crawfurdia* | *poilanei* | Hul | Favre 200 | Fansipan, Lào Cai | Vietnam | LZ |
| *Sinogentiana* | *souliei* | (Franch.) Adr.Favre & Y.M.Yuan | Fu2016143-1 | Lijiang, Yunnan | China | HLY |
| *Sinogentiana* | *striata* | (Maxim.) Adr.Favre & Y.M.Yuan | Fu2017353-3 | Guide, Qinghai | China | HLY |
| *Tripterospermum* | *championii* | Gardner | Favre 001 | Gunung Brinchang, Perak | Malaysia | KUN |
| *Tripterospermum* | *luteoviride* | (C.B.Clarke) J.Murata | Favre 015 | Gangtok, Sikkim | India | LZ |

**TABLE A2** List of species included in Figure 2 and Figure 3.

| ID | Species |
| --- | --- |
| sect. *Chondrophyllae*_1 | *Gentiana aristata* |
| sect. *Chondrophyllae*_2 | *Gentiana producta* |
| sect. *Chondrophyllae*_3 | *Gentiana crassuloides* |
| sect. *Chondrophyllae*_4 | *Gentiana haynaldii* |
| sect. *Chondrophyllae*_5 | *Gentiana cuneibarba* |
| sect. *Cruciata* | *Gentiana cruciata* |
| sect. *Pneumonanthe* | *Gentiana scabra* |
| sect. *Isomeria* | *Gentiana szechenyii* |
| sect. *Calathianae* | *Gentiana bavarica* |
| sect. *Gentiana* | *Gentiana lutea* |
| sect. *Ciminalis* | *Gentiana clusii* |
| sect. *Phyllocalyx* | *Gentiana phyllocalyx* |
| sect. *Kudoa* _1 | *Gentiana hexaphylla* |
| sect. *Kudoa* _2 | *Gentiana veitchiorum* |
| sect. *Kudoa* _3 | *Gentiana altigena* |
| sect. *Frigidae* | *Gentiana atuntsiensis* |
| sect. *Microsperma* | *Gentiana yunnanensis* |
| sect. *Monopodiae_*1 | *Gentiana davidii* |
| sect. *Monopodiae_2* | *Gentiana sikkimensis* |
| *Crawfurdia* | *Crawfurdia campanulacea* |
| *Kuepferia* | *Kuepferia damyonensis* |
| *Metagentiana* | *Metagentiana rhodantha* |
| *Sinogentiana*_1 | *Sinogentiana striata* |
| *Sinogentiana*_2 | *Sinogentiana souliei* |
| *Swertia* | *Swertia mussotii* |
| *Tripterospermum* | *Tripterospermum championii* |
